# Gasdermin-B Pro-Tumor Function in Novel Knock-in Mouse Models Depends on the *in vivo* Biological Context

**DOI:** 10.3389/fcell.2022.813929

**Published:** 2022-02-24

**Authors:** David Sarrio, Alejandro Rojo-Sebastián, Ana Teijo, María Pérez-López, Eva Díaz-Martín, Lidia Martínez, Saleta Morales, Pablo García-Sanz, José Palacios, Gema Moreno-Bueno

**Affiliations:** ^1^ Departamento de Bioquímica, Instituto de Investigaciones Biomédicas “Alberto Sols” (CSIC-UAM), Universidad Autónoma de Madrid (UAM), Madrid, Spain; ^2^ Centro de Investigación Biomédica en Red de Cáncer (CIBERONC), Madrid, Spain; ^3^ Fundación MD Anderson Internacional, Madrid, Spain; ^4^ Servicio de Anatomía Patológica, Hospital Ramón y Cajal, Universidad de Alcalá, IRYCIS, Madrid, Spain

**Keywords:** Gasdermins, tumorigenesis, Cancer Progression, novel mouse models, pyroptosis

## Abstract

Gasdermins (*GSDM*) genes play complex roles in inflammatory diseases and cancer. Gasdermin-B (*GSDMB*) is frequently upregulated in human cancers, especially in HER2-amplified breast carcinomas, and can promote diverse pro-tumor functions (invasion, metastasis, therapy-resistance). In particular, the *GSDMB* shortest translated variant (isoform 2; GSDMB2) increases aggressive behavior in breast cancer cells. Paradoxically, GSDMB can also have tumor suppressor (cell death induction) effects in specific biological contexts. However, whether GSDMB has inherent oncogenic, or tumor suppressor function *in vivo* has not been demonstrated yet in preclinical mouse models, since mice lack *GSDMB* orthologue. Therefore, to decipher *GSDMB* cancer functions *in vivo* we first generated a novel knock-in mouse model (R26-GB2) ubiquitously expressing human *GSDMB2*. The comprehensive histopathological analysis of multiple tissues from 75 animals showed that nucleus-cytoplasmic GSDMB2 expression did not clearly affect the overall frequency nor the histology of spontaneous neoplasias (mostly lung carcinomas), but associated with reduced incidence of gastric tumors, compared to wildtype animals. Next, to assess specifically the GSDMB2 roles in breast cancer, we generated two additional double transgenic mouse models, that co-express GSDMB2 with either the HER2/NEU oncogene (R26-GB2/MMTV-NEU mice) or the Polyoma middle-T antigen (R26-GB2/MMTV-PyMT) in breast tumors. Consistent with the pro-tumor effect of GSDMB in HER2+ human breast carcinomas, R26-GB2/MMTV-NEU GSDMB2-positive mice have double breast cancer incidence than wildtype animals. By contrast, in the R26-GB2/MMTV-PyMT model of fast growing and highly metastatic mammary tumors, GSDMB2 expression did not significantly influence cancer development nor metastatic potential. In conclusion, our data prove that GSDMB2 *in vivo* pro-tumor effect is evidenced only in specific biological contexts (in concert with the HER2 oncogene), while GSDMB2 alone does not have overall intrinsic oncogenic potential in genetically modified mice. Our novel models are useful to identify the precise stimuli and molecular mechanisms governing GSDMB functions in neoplasias and can be the basis for the future development of additional tissue-specific and context-dependent cancer models.

## Introduction

The Gasdermins (GSDMs, named after their Gastric and Dermal expression) are cytosolic proteins of around 50 KDa (Tamura et al., 2007; Saeki and Sasaki, 2012) that have been functionally involved in the genesis and development of cancer (Loveless et al., 2021; Sarrió et al., 2021) and multiple diseases (Broz et al., 2020; Liu et al., 2021). The GSDM family comprises six genes in the human genome (Tamura et al., 2007; Saeki and Sasaki, 2012; de Schutter et al., 2021): *GSDMA* and *GSDMB* (which are both located in the 17q21.1 region), *GSDMC* and *GSCMD* (in 8q24); *GSDME/DFNA5* (7p15.3) and *DFNB59/PJVK* (2q31.2). Mice have ten *GSDM* genes but *GSDMB* (also known as *GSDML* and *PRO2521*) is the only *GSDM* member that is not present in the mouse or rat genomes (Tamura et al., 2007). Each GSDM is expressed in diverse tissues in a cell-type specific way (Broz et al., 2020; de Schutter et al., 2021).

The diverse biological functions of GSDM proteins have recently started to emerge. Multiple studies indicate that all family members, except PJVK, can produce a lytic and pro-inflammatory cell death mechanism termed pyroptosis, while specific GSDMs in precise biological contexts can also provoke necrosis, apoptosis, NETosis mitochondrial damage, or autophagy (Broz et al., 2020; Liu et al., 2021). These cell-death promoting functions are normally auto-inhibited through the intramolecular interaction of GSDMs N-terminal (NT, pore-forming) and C-terminal (CT, inhibitory) domains (Ding et al., 2016; Wang and Ruan, 2021). Under certain stimuli and circumstances, the NT is exposed or released, via specific protease cleavage (caspases, granzymes and others) and produces cell death generally through the formation of NT membrane pores (Ding et al., 2016; Wang and Ruan, 2021) and mitochondrial damage (Lin et al., 2015; Shi et al., 2015; de Vasconcelos et al., 2019; Rogers et al., 2019), among other mechanisms. The release of pro-inflammatory molecules during pyroptosis induces robust reaction of the immune system (Broz et al., 2020; Liu et al., 2021). Through their pro-cell death activities and other functions, the GSDMs play a key role in the pathogenesis of several inflammatory or infectious diseases, among others (Broz et al., 2020; Li et al., 2021; Liu et al., 2021). In cancer, the involvement of GSDMs in tumor progression and clinical behavior is intricate, as GSDMs can promote either pro-tumor or anti-tumor effects, depending on the context (Sarrió et al., 2021). On one hand, the activation of GSDM-mediated tumor pyroptosis can lead to immune anti-tumor response, and thus GSDMs can act as tumor suppressor (Wang et al., 2020; Zhang et al., 2020; Zhou et al., 2020; Loveless et al., 2021). On the other hand, GSDM over-expression can mediate pro-tumor effects, and sometimes associate with unfavorable cancer prognosis (Hou et al., 2020; Sarrió et al., 2021). In particular, GSDMB is frequently expressed (mRNA/protein) in esophageal, gastric, colon, liver, breast, cervical and bladder cancers (Carl-McGrath et al., 2008; Sun et al., 2008; Komiyama et al., 2010; Hergueta-Redondo et al., 2014, 2016; He et al., 2021). GSDMB overexpression associates with advanced disease or poor prognosis in breast, oral and gastric cancer (Nguyen et al., 2007; Komiyama et al., 2010; Hergueta-Redondo et al., 2014, 2016), and increases invasive and/or metastatic behavior in breast and bladder cancer cells (Hergueta-Redondo et al., 2014; Molina-Crespo et al., 2019; He et al., 2021). Moreover, *GSDMB* gene is frequently co-amplified with *ERBB2/HER2* oncogene in breast and gastro-esophageal carcinomas (Saeki et al., 2009; Hergueta-Redondo et al., 2016), and in HER2+ breast cancers *GSDMB* upregulation promotes tumor aggressiveness and resistance to anti-HER2 therapies (Hergueta-Redondo et al., 2016). Interestingly, we demonstrated that GSDMB is a novel therapeutic cancer target, since its multiple pro-cancer functions in HER2+/GSDMB + breast cancer cells were reduced *in vitro* and *in vivo* by the intracellular delivery of a GSDMB antibody through biocompatible nanocapsules (Molina-Crespo et al., 2019). Altogether, these data indicate that GSDMB could act as an oncogene. However, it could also have antitumor effect in certain biological contexts. Specifically, in cancer cells xenografted in immunocompetent mice, the GSDMB intrinsic pyroptotic function could be activated via immunocyte-derived Granzyme A (GZMA). GZMA cleaves and activates GSDMB NT in cancer cells subsequently producing tumor pyroptosis and cancer regression (Zhou et al., 2020).

Furthermore, there are at least four GSDMB translated isoforms (ENSEMBL:ENSG00000073605) that differ by the alternative usage of exons 6 and 7 (encoding residues located in the protein inter-domain (Chao et al., 2017)): isoform-1 (NM_001042471.1; lacks exon 6, protein of 403 aminoacids–aas- and 45.8 KDa), isoform-2 (NM_018530.2; lacking exons 6 and 7, 394 aas and 45 KDa), isoform-3 (NM_001165958.1; “full-length” protein of 416 aas and 47.4 KDa) and isoform 4 (NM_001042471.1; without exon 7, 407 aas and 46.5 KDa). The differential isoform expression in cells and tissues could lead to distinct functional consequences in normal and pathological contexts, such as inflammatory diseases (Morrison et al., 2013; Panganiban et al., 2018) and cancer (Carl-McGrath et al., 2008; Sun et al., 2008; Hergueta-Redondo et al., 2014; Lutkowska et al., 2017). In this sense, we previously showed that overexpression of either isoform 1 or 2 increases motility and invasion *in vitro*, but only the isoform 2 (the shortest transcript; hereafter referred to as GSDMB2) promotes tumor growth and metastasis of MCF7 breast cancer xenografts (Hergueta-Redondo et al., 2014). This data suggested an increased pro-tumor potential for GSDMB2.

However, whether GSDMB has inherent oncogenic potential or affect cancer development and progression *in vivo* has not been tested yet in preclinical mouse models since mice lack *GSDMB* orthologue. To answer these key questions, in this work we first generated a novel knock-in (KI) mouse model (R26-GB2) ubiquitously expressing human *GSDMB2* transcript. Then we created two novel double transgenic mouse models, that co-express GSDMB2 with either the HER2/NEU oncogene (R26-GB2/MMTV-NEU mice) or the Polyoma middle-T antigen (R26-GB2/MMTV-PyMT) in metastatic breast tumors. In these models we performed the first comprehensive *in vivo* study of GSDMB effects on tumor initiation and development.

## Materials and Methods

### Animals and Ethics Statement

All mouse studies were performed in agreement with the procedures and protocols that have been approved by the internal committees of ethical and animal welfare of the Institutions (Universidad Autónoma de Madrid and Instituto de Investigaciones Biomédicas Alberto Sols-CSIC) and the local authorities (Comunidad de Madrid, PROEX424/15 and PROEX 235.6/20). The procedures comply with the European Union (Directive 2010/63/UE) and Spanish Government guidelines (Real Decreto 53/20133). All animals were housed in the IIBm animal facility within the same room under standard conditions. The study adheres to the ARRIVE guidelines for the design, analysis, and reporting of animal research.

The commercial mouse strains B6.FVB-Tg (EIIa-Cre)C5379Lmgd/J, FVB/N-Tg (MMTV-PyVT)634Mul/J (Guy et al., 1992a), and FVB/N-Tg (MMTVneu)202Mul/J (Guy et al., 1992b) were purchased from JaxMice. The FVB/NCrl strain was purchased from Charles River. The generation of four novel animal models (R26-STOP-GB2, R26-GB2, R26-GB2/MMTV-PyMT and R26-GB2/MMTV-NEU) is described below.

#### Generation of conditional GSDMB2 Knock-in mice (R26-STOP-GB2)

**FIGURE 1:**
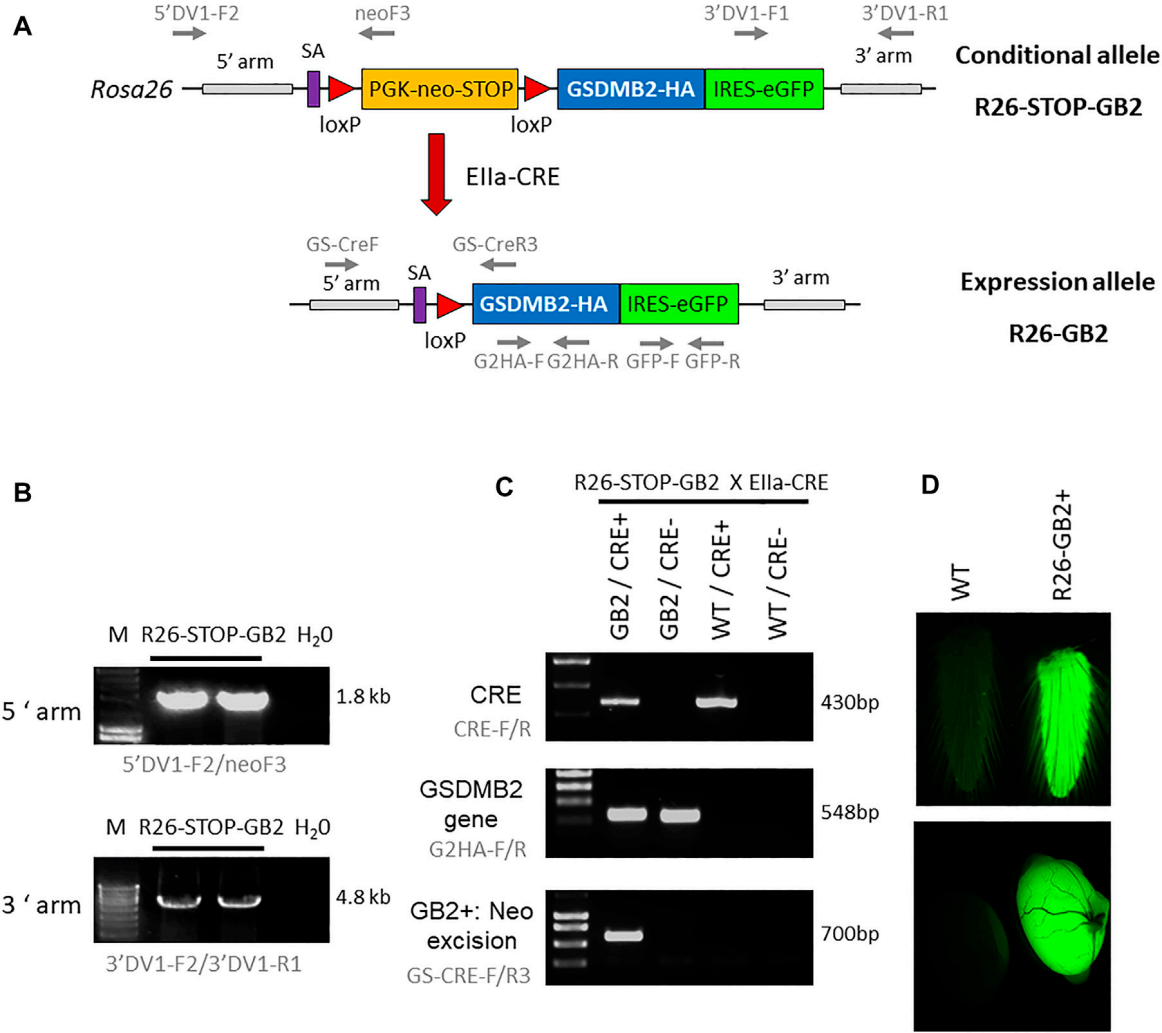
Generation of knock-in mouse models harboring human GSDMB isoform 2 transcript within the ROSA26 locus. **(A)** Schematic representation of the GSDMB isoform 2 (GB2) targeted floxed allele (top, conditional model) and the expression allele (bottom) within the ROSA26 (R26) locus. The construct contains a splicing acceptor signal (SA), the PGK-neomycin-STOP cassette flanked by LoxP sites, the human GSDMB2 isoform 2 cDNA sequence (GB2) fused with HA tag, followed by the IRES-GFP reporter gene. After crossing with EIIa-Cre strain (red arrow), the Cre-mediated excision of the PGK-neomycin-stop element allows the ubiquitous expression of the GB2-HA/GFP tandem under the control of the ROSA26 promoter (R26-GB2). The primer pairs for PCR analyses are also detailed (gray arrows). **(B)** Diagnostic PCR analysis of positive ES cell clones showing the proper insertion of the recombinant R26-STOP-GB2 allele. H20, Negative control. **(C)** Examples of genotyping PCR analysis (primers in gray), demonstrating the excision of neo-stop cassete in Cre+/GB2 mice. **(D)** Ubiquitous expression of the transgenes is verified by GFP fluorescent emission of fresh tail skin (top) and testes (bottom) from WT and GSDMB-positive R26-GB2 mice. Full-length gels are presented in Supplementary Figure S1.

We generated, in collaboration with the CNIO Transgenic mice Service, mice harboring human GSDMB isoform 2 transcript (NM_018530.2) essentially as previously reported (Nyabi et al., 2009; Martin et al., 2015). Using gene-targeting technology, we inserted by homologous recombination into the endogenous *ROSA26* (R26) locus a construct containing a loxP-flanked PGK-neomycin-STOP cassette (Nyabi et al., 2009) followed by the human GSDMB isoform 2 cDNA (GSDMB2) fused with the HA-tag sequence (Figure 1A). The construct also contains an IRES-sequence followed by GFP gene reporter, which helps in the identification of the Knock-in (KI) animals (Figure 1A). Expression of the construct is under the control of endogenous *Rosa26* promoter, which allows ubiquitous and moderate levels of expression of the transgene (Nyabi et al., 2009). The targeting vector for the homologous recombination was generated by the Gateway cloning DNA technology using the pEntry plasmid harboring GSDMB2-HA cDNA and the pROSA26-DV vector, as reported previously (Martin et al., 2015). Recombinant clones were sequence-verified using the S1F (5′-ATC​ATG​TCT​GGA​TCC​CCA​TC-3′) and S2R (5′-GGG​GCG​GAA​TTC​GAT​ATC​AAG-3′) primers. The targeting construct was then electroporated into ES cells (C57BL6x129 background; CNIO Transgenic Mice Service), and positive ES clones harboring the construct in the correct orientation were detected by diagnostic PCR (conditions detailed in Supplementary Table S1). Two positive ES clones were used for aggregation with CD1 embryos (CNIO Transgenic mice Service) obtaining 18 male chimeras (all >80% chimerism). After crossing with FVB wildtype (WT) female mice, the correct transmission of the transgene was demonstrated by two PCR tests (Figures 1B,C): a) insertion of the transgene into 3′- and 5′-arm; b) presence of GSDMB-HA transgene and GFP gene. PCR conditions are detailed in Supplementary Table S1; Uncropped PCR gels are provided in Supplementary Figure S1. After confirmation of correct transgene insertion by PCR, we derived one mouse strain of conditional GSDMB2 expression, referred to as R26-STOP-GB2. This strain has mixed genetic background (C57BL6x129 from the ES, CD1 from the embryo aggregation and crossed twice with FVB).

### Generation of Knock-in Mice with Ubiquitous Expression of GSDMB2-HA (R26-GB2)

To analyze *in vivo* the consequences of ubiquitous GSDMB2 expression in mice, conditional R26-STOP-GB2 male animals were crossed with female mice of the B6.FVB-Tg (EIIa-Cre)C5379Lmgd/J strain (JAXmice). Adenoviral EIIa promoter expression is restricted to oocytes and preimplantation stages of the embryo, and thus Cre-mediated recombination occurs in a wide range of tissues, including the germ cells that subsequently transmit the genetic modification to progeny. The deletion of the neo-STOP cassette by Cre permits the transcriptional expression, mediated by R26 promoter, of the bicistronic mRNA GSDMB2-HA-IRES-GFP (Figure 1A). To verify the correct excision of the neo cassette and the subsequent activation of the transgenes we designed a diagnostic PCR reaction that preferentially amplifies the excised allele in DNA obtained from tail skin (Figure 1C). PCR conditions are detailed in Supplementary Table S1. Uncropped PCR gels are provided in Supplementary Figure S1. In these mice, GFP light emission, used as a readout of transgene expression, was clearly detected in some fresh tissues, such as tail or testes (Figure 1D) using a Leica MZ10F magnifier. After validation of transgene ubiquitous expression, we crossed heterozygous animals to remove the Cre recombinase and to obtain a line expressing germline GSDMB2-HA-GFP in all tissues. This mouse model, named R26-GB2, with mixed background was crossed two times with the FVB/NCrl strain (Charles River) to ensure that it contained at least 50% FVB genetic background.

### Generation of Breast Cancer Mouse Models Expressing GSDMB2-HA With Either the HER2/NEU Oncogene (R26-GB2/MMTV-NEU) or PyMT Oncogene (R26-GB2/MMTV-PyMT)

The R26-GB2 model was crossed with either the FVB-MMTV-NEU-202Mul/J or FVB-MMTV-PyMT strains (JaxMice). The mammary glands of female animals from the MMTV-NEU or MMTV-PyMT express the inactivated rat neu oncogene (Guy et al., 1992b) or the Polyoma Middle T antigen (Guy et al., 1992a), respectively, under the regulation of the MMTV (Mouse Mammary Tumor Virus) promoter. Fifty per cent of female MMTV-NEU homozygous mice develop spontaneous invasive breast carcinomas by 7 months of age and metastatic lung colonization occurs in 72% of them after 8 months of age (Guy et al., 1992b). Female MMTV-PyMT mice develop spontaneous invasive breast carcinomas and metastatic lung colonization by 14 weeks of age and (Guy et al., 1992a). To generate R26-GB2/MMTV-NEU or R26-GB2/MMTV-PyMT double transgenic animals, female homozygous R26-GB2 animals were crossed with male homozygous MMTV-NEU or MMTV-PyMT mice. Then, male mice heterozygous for NEU or PyMT and GSDMB2 were crossed with female heterozygous R26-GB2 animals. Genotyping of the NEU and PyMT oncogenes was performed as described in Supplementary Table S1.

### Phenotypic and Histological Characterization of R26-GB2 Model: Study of Spontaneous Tumorigenesis

Heterozygous R26-GB2 animals were crossed to obtain at least 18 animals from each of the genotypes. A total of 80 mice (42 males, 38 females) corresponding to the three GB2 genotypes (WT, *n* = 27; GB2+/−, *n* = 34 and GB2+/+, *n* = 19) were studied up to 18 months of age. Five animals died spontaneously, and no necropsy could be performed, thus were excluded from the histological analyses. Mice were monitored weekly for the appearance of tumor masses (in any part of the body) or other pathological signs (outcome). Animals were sacrificed when they reached 18 months of age or showed any of the criteria for early termination (scored as 4) (Orellana-Muriana et al., 2012). These criteria include: tumors >15 mm, ulceration or infection of the tumors, body weight loss >20%, enlarged lymph nodes, or extensive skin ulceration, among others (Orellana-Muriana et al., 2012). Animals were euthanized in a CO_2_ chamber (fill rate of 30% of the chamber volume per minute) and necropsy was performed immediately. We extracted all organs with macroscopic signs of cancer or other pathologies, as well as other selected organs with normal appearance. Tissues were fixed in 10% formalin and embedded in paraffin blocks. For histologic examination, Hematoxylin-Eosin-stained (H&E) tissue sections were analyzed by two Pathologists (ARS and AT). A total of 328 tissue sections were reviewed (median = 3 organs per mice; minimum 1 and maximum of 14).

### Study of Breast Tumorigenesis and Metastatic Potential in R26-GB2/MMTV-NEU or R26-GB2/MMTV-PyMT Mice

Seventy-two R26-GB2/MMTV-NEU female mice, all heterozygous for NEU (21 GB2+/+, 26 GB2+/- and 25 WT) were monitored once a week for a mean follow-up time of 15 months. Three GB2+/- tumor-bearing animals were found dead, and necropsy could not be performed (tumor tissue and lungs were decomposed). Thus, histological data was recorded in 69 mice. In the R26-GB2/MMTV-PyMT model we used 27 PyMT heterozygous female mice (10 GB2+/+ and 17 WT) and breast tumor development was monitored 2–3 times a week until animals reached 15 weeks of age. Animals were euthanized when they either developed a mammary tumor bigger than 1.5 cm long, reached the endpoint date without tumor or showed any of the criteria for early termination (scored as 4) (Orellana-Muriana et al., 2012). Mice were sacrificed by CO_2_ inhalation (fill rate of 30% of the chamber volume per minute) and all mammary glands with breast tumors were extracted (mean 1.5 tumors/mice in the R26-GB2/MMTV-NEU and 5.6 tumors/mice in the R26-GB2/MMTV-PyMT model), washed in 1x PBS, measured with caliper, and weighted on a scale. The biggest tumor of each mouse was selected, and half of the cancer tissue was processed for subsequent histological analysis by two pathologists (as described above). The rest of the tissue was quickly frozen in dry-ice and stored at −80°C. We analyzed the cancer incidence, latency (time until detection of palpable tumor), number and weight of tumors. Moreover, to assess metastatic potential the whole lungs were extracted and processed for subsequent paraffin-embedding. Then, whole lungs were serially sectioned into 5 µm-thick tissue sections using a microtome (Leica RM 2255). From these slides, we selected four sections separated by 100 µm in depth and stained with H&E. Thus, the analyses of these four combined sections covered >400 µm in depth. The sum of metastatic foci observed in the four slides was calculated. Metastatic lesions that appear repeatedly in two or more slides were counted once.

### Immunohistochemical Analysis

GSDMB2-HA expression was analyzed by immunohistochemistry in 5 µm-thick tissue sections using rat anti-HA (1:200; 3F10, ROCHE) or mouse monoclonal anti-GSDMB [1:10, (Hergueta-Redondo et al., 2016)], following standard methods. HER2/NEU membrane receptor was detected with rabbit anti-HER2 (prediluted; A0485, Dako). Tumor proliferation in the R26-GB2/MMTV-PyMT animals was assessed by PCNA (proliferating cell nuclear antigen) using the MAB424R antibody (1:10,000; clone p10, Millipore). Briefly, after an antigen-retrieval step (Leica Bond ER solution-1, citrate buffer 10 mM pH 5.9–6.1) the primary antibodies were incubated for 1 h at RT, followed by secondary-HRP antibody incubation. The staining was revealed by DAB standard Leica procedure. In negative controls, the primary antibodies were omitted. Immunohistochemical images were taken from representative samples with an Axiophot (Zeiss) microscope coupled with a color DP70 (Olympus camera), using the Olympus DP controller software. For immunofluorescence analysis, secondary goat anti-rat IgG-Alexa 547 (1:1,000, Molecular probes) was incubated for 1 h at RT. Slides were stained with 1:10,000 DAPI (4′,6-diamino-2- fenilindol, Molecular Probes), mounted with Prolong Diamond Antifade Mountant (Molecular Probes) and analyzed by confocal microscopy (LSM710, Zeiss).

### Analysis of GSDMB2-HA-GFP Tissue Expression by Western Blot

Six R26-GB2 mice (3 males, 3 females) from each of the GB2 genotypes (WT, GB2+/-, GB2+/+) were sacrificed at 20 weeks of age. Sixteen organs were removed, chopped and immediately stored at -20°C. Tumors from 13 R26-GB2/MMT-NEU and 12 R26-GB2/MMT-PyMT were also collected. Tissues were homogenized in 50–200 µL lysis buffer (0.1 M NaCl, 0.05 M Tris HCl pH 7.9, 5 μM MgCl_2_, 5 μM CaCl_2_, 2% SDS supplemented with 1x protease inhibitor cocktail, ROCHE) by sonication on ice. Lysates were clarified by centrifugation (10.000 rpm, 5 min) and quantified by the BCA method (Pierce). Fifty µg total proteins/per sample were loaded on 10% SDS-PAGE gels. WB were performed by standard methods using rat anti-HA (1:1,000; clone 3F10, ROCHE), rabbit anti-GFP (1:2000; A6455, Molecular Probes) and mouse anti-GAPDH (1:50,000; 6C5, Calbiochem). As positive control, we used a sample of MCF7 cells expressing GSDMB2-HA (Hergueta-Redondo et al., 2014) and GFP constructs. Uncropped Western blots are provided in Supplementary Figure S1.

### Flow Cytometry

To evaluate the proportion of white blood cells from R26-GB2 mice expressing the GSDMB-HA-GFP transgenes we analyzed GFP emission by Flow Cytometry (Cytomics FC 500MPL, Beckman Coulter). Total leukocyte cells, not any specific subpopulation, were analyzed. Peripheral blood from WT and GB2+/+ mice was extracted and processed following the method reported before (Canesin et al., 2015).

### Statistical Analyses

Data was obtained from all available animals in the study (each mouse corresponds to a data point) and, unless otherwise specified, no data points were excluded from the analyses. The normal distribution of the continuous data was confirmed by the Kolmogorov–Smirnov test. Statistical analyses were performed using GraphPad 6.0 (GraphPad Software, Inc.) using Chi^2^ or Fisher’s exact tests to assess differences in categorical variables, and ANOVA or Student t-test for continuous variables. A *p value* <0.05 was considered as statistically significant.

## Results

### Generation of the Knock-in Mouse Model Ubiquitously Expressing GSDMB2-HA (R26-GB2)

To assess *in vivo* the cancer roles of GSDMB isoform 2 (GSDMB2), a transcript that promotes invasive and metastatic behavior of MCF7 breast cancer cells (Hergueta-Redondo et al., 2014), we first generated a conditional KI model (R26-STOP-GB2) and then derived the model (R26-GB2) ubiquitously expressing GSDMB2-HA and GFP transgenes in the whole body of the animal (Materials and Methods and Figures 1A–D).

Homozygous (hereafter referred to as GB2+/+) and heterozygous (GB2+/-) R26-GB2 mice are viable and fertile, reproduce normally, and female mice can nurse their litter naturally. In crossings between heterozygous animals, the transgene is transmitted with expected frequencies of the Mendelian inheritance. Transgenic mice do not show evident morphological and developmental alterations or signs of abnormal behavior. GB2+/+ mice tend to have slightly higher body weight, especially in males, than WT animals but the differences do not reach statistical significance (Supplementary Figure S2).

### Expression and intracellular Localization of GSDMB2-HA in Tissues

**FIGURE 2:**
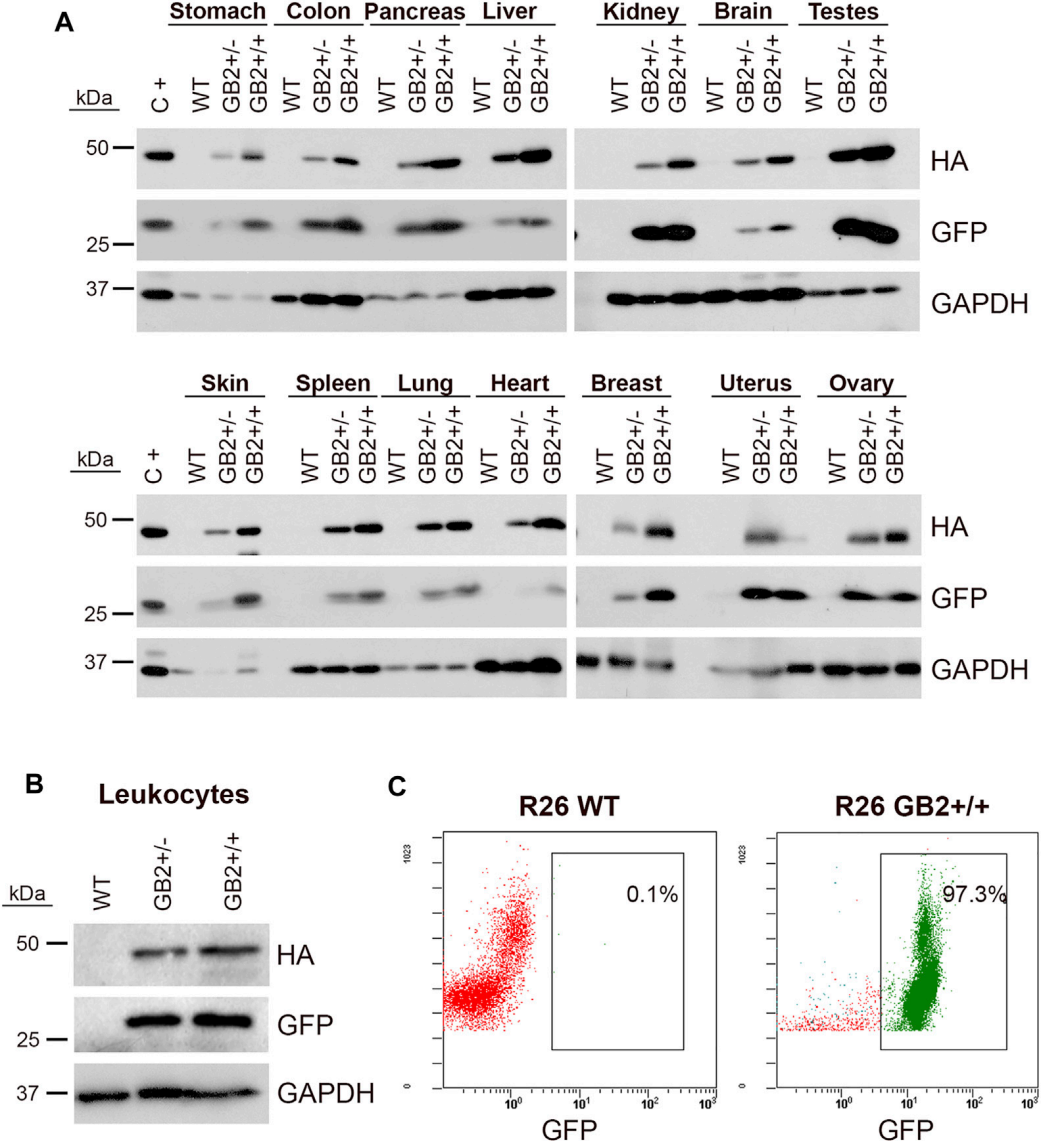
Ubiquitous expression of GSDMB2-HA and GFP in the R26-GB2 mouse model. **(A)** Representative western blot analysis in different tissues from GB2 (+/- heterozygous; +/+ homozygous) and WT (control) littermate mice. GAPDH was used as a loading control. C+, MCF7 exogenously expressing GSDMB2-HA and GFP genes were used as a positive control. **(B–C)** Expression of GSDMB2-HA and GFP transgenes by WB **(B)** and GFP by flow cytometry **(C)** in whole blood leukocytes from R26-GB2 mice. Full-length blots are presented in Supplementary Figure S1.

In humans, *GSDMB* mRNA is detected in multiple organs and tissues, including the digestive tract, respiratory system, immune cells, reproductive organs, and breast tissue among others (Broz et al., 2020). Using immunohistochemistry, GSDMB protein shows different patterns of expression and intracellular localization depending on the tissue and antibody used (Carl-McGrath et al., 2008; Sun et al., 2008; Das et al., 2016; Hergueta-Redondo et al., 2016; Zhou et al., 2020; Protein atlas, https://www.proteinatlas.org/) (Supplementary Table S2). To test GSDMB2 broad expression in our model, first we verified by Western Blot the specific detection of GSDMB2-HA and GFP proteins in 14 organs from male and female GB2 mice (Figure 2A). Expression of the transgenic construct in peripheral blood leukocytes was additionally demonstrated by WB and FACs analysis (Figures 2B,C), where more than 90% of cells showed GFP expression (Figure 2C).

**FIGURE 3:**
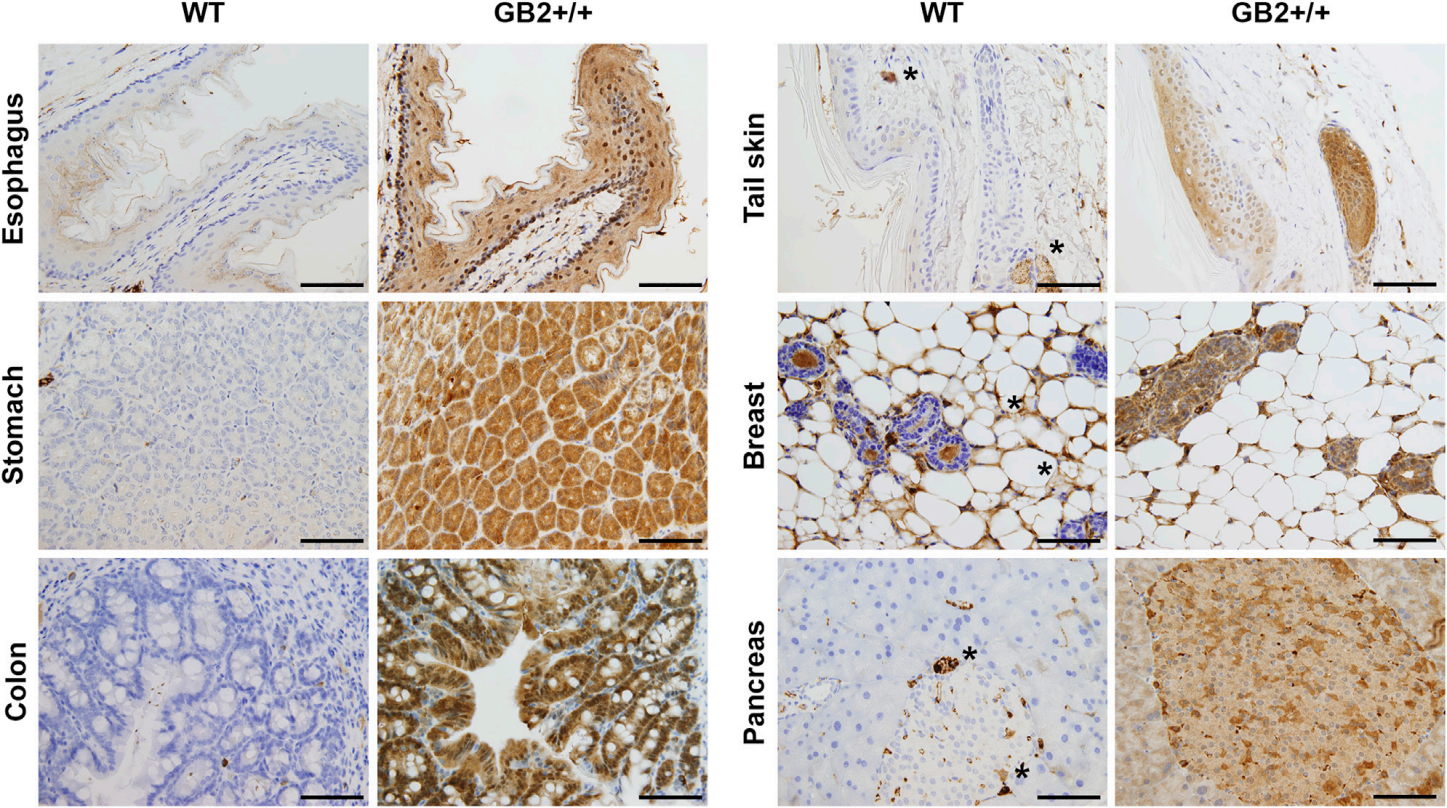
Immunohistochemical expression of GSDMB2-HA in different tissues of the R26-GB2 mouse model. Representative images of tissues from homozygous (GB2+/+) and control (WT) mouse littermates. * Unspecific staining. Scale bar, 100 µm.

Next, we analyzed GSDMB2-HA expression and subcellular localization in diverse GB2+ and WT tissues by immunohistochemistry using an anti-HA antibody. GSDMB2 showed mainly cytoplasmic localization in some tissues, such as breast, pancreas, or liver, while nucleo-cytoplasmic staining was typically seen in specific tissues/cell types (Figure 3, Supplementary Figure S3; Supplementary Table S2). Nuclear staining was particularly strong in the squamous epithelia of the esophagus, skin epidermis, hair follicles and sebaceous glands, as well as colon epithelia (Figure 3 **)** and testicles (Supplementary Figure S4), among others (Supplementary Table S2). In all tissues, GSDMB2 showed the same staining pattern in heterozygous and homozygous mice, but in GB2+/+ animals nuclear accumulation was more evident. To confirm the nuclear-cytoplasmic localization, additional staining with our anti-GSDMB monoclonal antibody (Hergueta-Redondo et al., 2016) was performed in testis. Both HA and GSDMB antibodies showed the same expression pattern (Supplementary Figure S4A). Moreover, the nuclear localization in this tissue was confirmed further by immunofluorescence and confocal microscope analysis (Supplementary Figure S4B). Overall, the nuclear-cytoplasmic pattern of GSDMB2-HA in mice resembles to that observed in the corresponding human tissues (Supplementary Table S2). Furthermore, the differences found in the intracellular localization patterns among tissues could indicate that GSDMB2 has possibly distinct functions depending on the cell context.

### Effect of GSDMB2 on Spontaneous Tumorigenesis *in vivo*

GSDMB over-expression occurs in diverse tumor types and promotes multiple pro-tumor functions in human breast and bladder cancer cells (Sarrió et al., 2021), suggesting that GSDMB could have intrinsic oncogenic properties. To investigate if GSDMB2 expression has *in vivo* spontaneous tumorigenic activity in any tissue we studied 80 mice for up to 18 months. Mice were monitored weekly for the appearance of tumor masses or other pathological signs and were sacrificed when they showed any of the criteria for early termination specified in Methods or reached 18 months of age. Five mice (all WT) were found dead and necropsy could not be performed, thus post-mortem analyses were done in 75 mice. The overall survival of all the animals (including mice found dead and those sacrificed according to early termination criteria) was similar among GB2+/-, GB2+/+ and WT mice (log-rank Mantel Cox test, *p* = 0.6). At necropsy, tumor formation was investigated in multiple tissues and organs, but macroscopic cancers were only frequently seen in the lungs and stomach (Table 1). In fact, the most common neoplasias observed (41% including all mice) were lung adenocarcinomas, which is consistent with the frequency of these spontaneous tumors in elder mice of the FVB background (Mahler et al., 1996). However, no significant differences in the frequency of lung tumors (Table 1) were observed between WT and GB2 mice (Chi^2^ test *p* = 0.20, considering the three genotypes; Fisher’s exact test *p* = 0.79 comparing WT versus the combination of GB2+/+ and GB2+/-; Fisher’s exact test *p* = 0.51 comparing WT versus GB2+/+). Moreover, most of these tumors were well-differentiated lung adenocarcinomas, and no differences in histological grade among the genotypes were observed (Supplementary Table S3).

**TABLE 1:** Frequency of spontaneous tumors in GSDMB2-HA knock-in mouse model (R26-GB2) and control (WT) mice.

Genotype and gender	N	Lung	Stomach	Others
WT Male	13	7 (54%)	3 (23%)	1 Lymphoma
WT Female	10	2 (20%)	1 (10%)	
**WT TOTAL**	**23**	**9 (39%)**	**4 (17%)**	
GB2+/-Male	17	9 (53%)	0	1 Hepatocarcinoma
GB2+/-Female	16	8 (50%)	0	
**GB2+/-Total**	**33**	**17 (52%)**	**0**	1 Lymphoma
GB2+/+ Male	9	2 (22%)	0
GB2+/+ Female	10	3 (30%)	0	1 Breast cancer
**GB2+/+ Total**	**19**	**5 (26%)**	**0**	
**All mice**	**75**	**31 (41%)**	**4 (5%)**	**4 (5%)**

GSDMB2 homozygous (GB2+/+), heterozygous (GB2+/-) and WT animals were generated by crossing parental heterozygous mice. Mice were monitored for up to 18 months of age and tissues with macroscopic tumors were analyzed. The bold text is the sum of the above rows.

**FIGURE 4:**
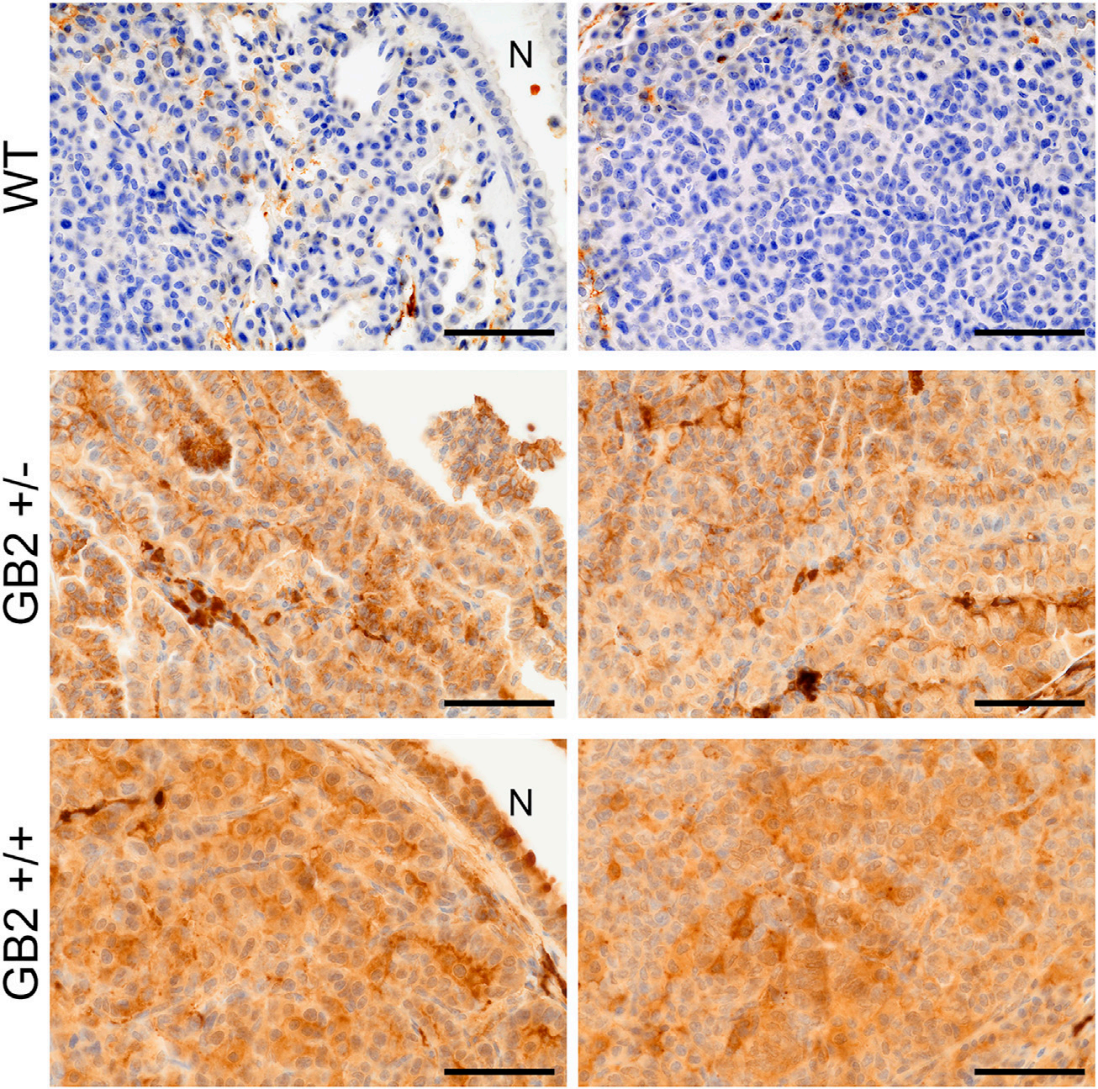
Immunohistochemical expression of GSDMB2-HA in spontaneous lung carcinomas from the R26-GB2 mouse model. Representative images of lung cancers from homozygous (GB2+/+), heterozygous (GB2+/-) and control (WT) mice. Note the stronger expression of GSDMB2-HA in GB2+/+ than GB2+/- cancer cells and the negative staining in the WT condition. N: normal lung bronchiole. Scale bar, 100 µm.

Next, to ensure that GSDMB2-HA protein was expressed in these lung tumors, we performed immunohistochemical analyses using an anti-HA antibody. In GB2+/+ and GB2+/-lungs we confirmed the diffuse cytoplasmic and focal nuclear staining (stronger in GB2+/+) of GSDMB2-HA in both carcinoma cells and the normal bronchioles (Figure 4). The positive staining with the C-terminal HA tag proves that the full-length GSDMB2 protein is expressed in tumor cells, but it does not have a clear impact on lung cancer development.

The second most frequent cancer type were gastric tumors. Interestingly, we observed that 17% WT mice (3 male and 1 female) developed macroscopic gastric carcinomas, but none of GB2 mice did (Table 1) (Chi^2^ test *p* = 0.008, considering the three genotypes; and Fisher’s exact test *p* = 0.007 comparing WT versus the combination of GB2+/+ and GB2+/−). This result suggests that GSDMB2 might reduce spontaneous gastric tumor formation.

Other types of cancer (e.g., breast cancer) were seldom observed in GB2 or WT animals (Table 1). Therefore, taking all cancers together (irrespective of the tissue of origin) there were no differences in spontaneous tumor frequency among the genotypes (Chi^2^ test *p* = 0.28, considering the three genotypes; Fisher’s exact test *p* = 0.33 comparing WT versus the combination of GB2+/+ and GB2+/−).

Since frequent tumors were only seen in lung and stomach, to assess further the effect of GSDMB2 in tumorigenesis, we focused our histological analyses on these organs. Therefore, we evaluated the presence of microscopic tumors or pre-malignant lesions in a series of tissue samples not showing macroscopic evidence of cancer (lung, *n* = 39; stomach, *n* = 30; Supplementary Table S4). No tumors were detected in these samples, and the frequencies of premalignant adenomatous lung hyperplasia, gastric adenomas/polyps, or chronic gastritis, a potential precursor of stomach cancer, were similar in WT and GB2 mice (Supplementary Table S4).

As a whole, these data imply that human GSDMB2 alone does not have a strong overall tumorigenic potential in mice, but it might have instead a potential suppressive effect of gastric carcinogenesis.

### Study of Breast Tumorigenesis and Progression in the R26-GB2/MMTV-NEU and R26-GB2/MMTV-PyMT Models

**FIGURE 5:**
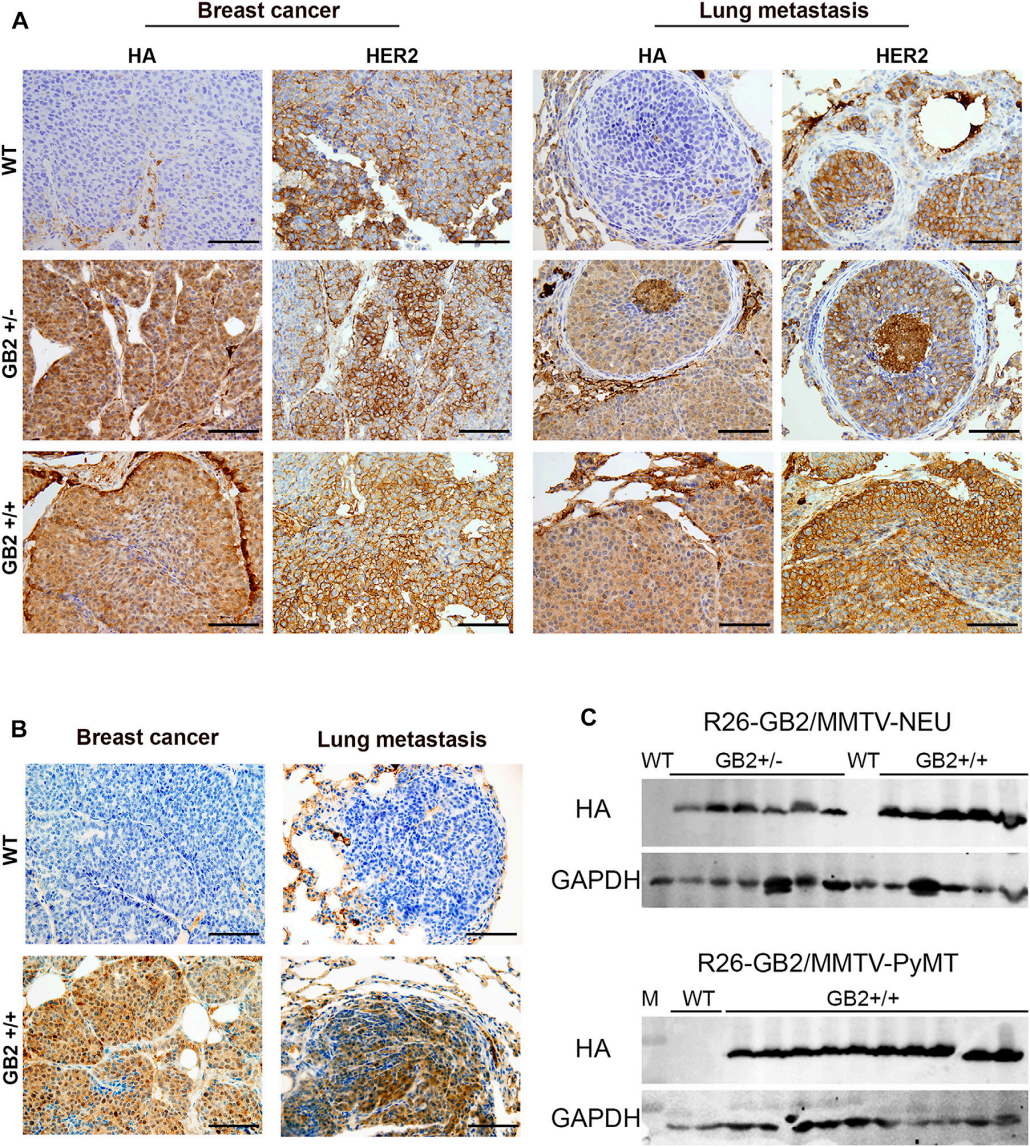
GSDMB2-HA expression in primary breast carcinomas and corresponding lung metastasis from R26-GB2/MMTV-NEU model and R26-GB2/MMTV-PyMT mice. **(A)** Representative images of the GSDMB2-HA and HER2 immunohistochemical expression in primary tumors and metastatic foci from WT, GB2+/- and GB2+/+ GB2/MMTV-NEU mice of 15 months age. **(B)** Representative images of the GSDMB2-HA staining from GB2+/+ and WT GB2/MMTV-NEU mice of 15 weeks age. Scale bar, 100 µm. **(C)** Comparison of GSDMB2-HA expression between tumors from the two models by Western blot. GAPDH was used as loading control.

**FIGURE 6:**
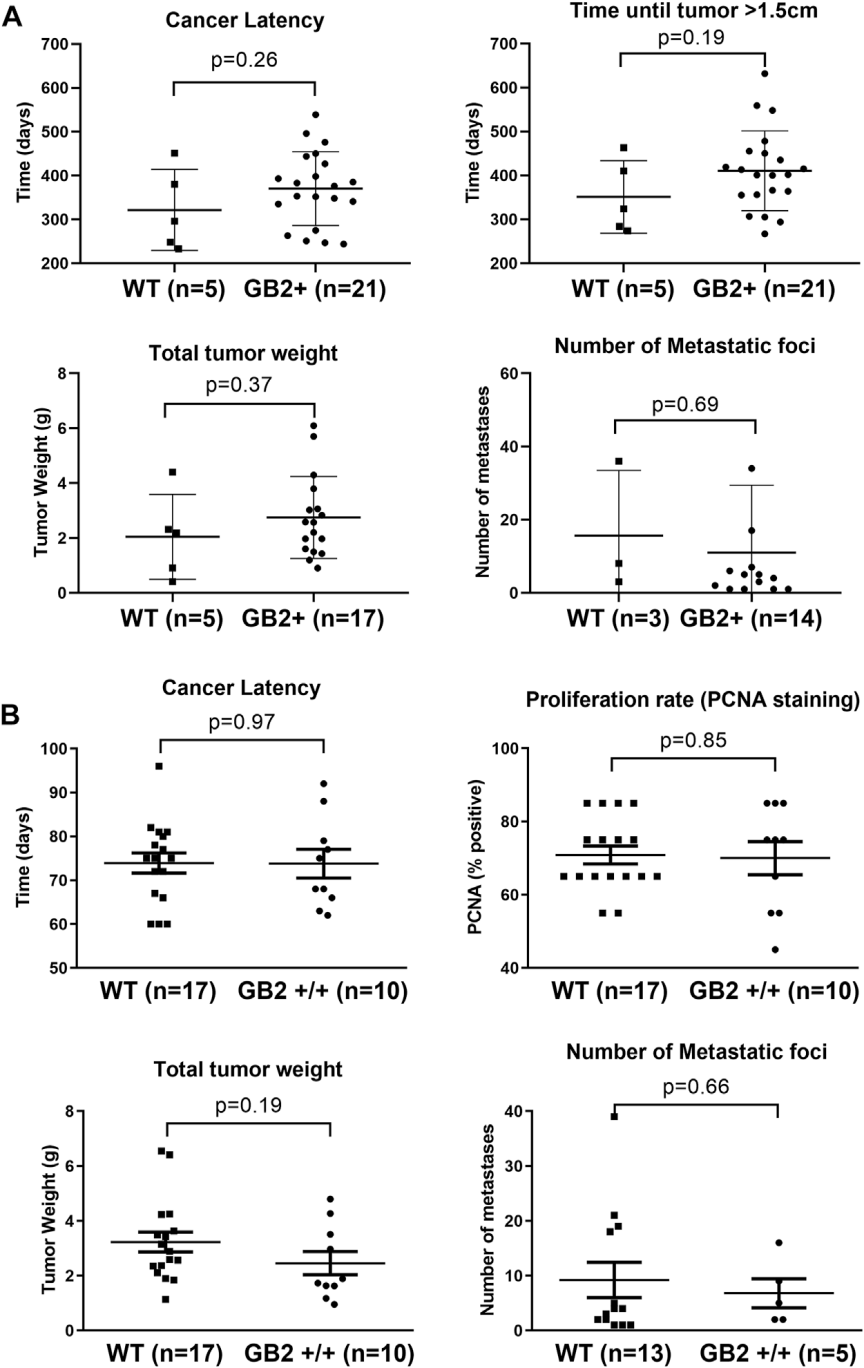
Effect of GSDMB2 on breast cancer generation and cancer progression in the R26-GB2/MMTV-NEU and R26-GB2/MMTV-PyMT mouse models. **(A)** Comparisons between GB2+ (GB2+/+ and GB2+/-combined) and WT mice of the GB2/MMTV-NEU model (median age 15 months). All mice were heterozygous for HER2/NEU oncogene. **(B)** Comparisons between GB2+/+ and WT mice of the GB2/MMTV-PyMT model (15 weeks of age). All mice were heterozygous for PyMT oncogene. Tumor latency: time until detection of palpable mammary tumors. Total tumor weight: Each data point represents the added weight of all tumors for each mouse. Proliferation rate: Percentage of PCNA-positive staining (only the biggest tumor of each mouse). Lung metastasis foci: number of metastatic lesions (only animals with metastasis). Graphs show all data points, mean values (line) and standard deviations (error bars). Statistical differences were tested by Student’s t-test.

While GSDMB over-expression in human breast cancers, specially the HER2 subtype, associates with disease aggressiveness (Hergueta-Redondo et al., 2014, 2016; Molina-Crespo et al., 2019), we only detected one case of spontaneous breast carcinoma in the GB2+/+ mice. This result suggests that the pro-tumor functions of GSDMB observed in human breast cancers may depend on the pre-activation of specific oncogenic stimulus. To test this hypothesis, we evaluated the effect of GSDMB2 expression on breast cancer generation and progression in concert with two different strong oncogenes, HER2/NEU (Guy et al., 1992b) or the PyMT (Guy et al., 1992a). To this end, we generated two double transgenic models, that expresses GSDMB2 ubiquitously (including the breast) with either NEU (R26-GB2/MMTV-NEU model) or PyMT (R26-GB2/MMTV-PyMT mice), which are expressed specifically in the mammary gland. In the R26-GB2/MMTV-NEU model, breast cancer development was monitored for a mean follow-up of 15 months in all experimental groups (WT, 457 days; GB2+/-, 459 days; GB2+/+, 448 days), while in R26-GB2/MMTV-PyMT mice we analyzed cancer development until 15 weeks of age. In both models, strong nucleus-cytoplasmic expression of GSDMB2 was observed in carcinomas cells within the primary tumor and in the lung metastases (Figures 5A,B). Moreover, overall GSDMB2 tumor expression was similar in both murine models (Figure 5C). In the context of HER2/NEU-induced tumors, a significant effect of GSDMB2 on breast cancer incidences was noted. In fact, GB2+/-or GB2+/+ mice exhibited double incidence compared to WT controls (Table 2). Moreover, while WT animals developed just one tumor per mice, GB2+/-or GB2+/+ usually developed multiple tumors (maximum 4, mean 1.67 tumors in GB2+/-or GB2+/+). Since GB2+/-and GB2+/+ showed equal cancer incidence and number of tumors per mice, these mice were analyzed as a single group “GB2+“. The cancer latency (time until palpable tumor detection) or the time until tumor reached >1.5 cm (sacrifice) tended to be shorter in WT than GB2+, but differences were not statistically significant (Figure 6). Moreover, no significant variations were observed in the mean tumor weight between groups (Figure 6). At the histological level, all tumors showed high-grade solid invasive pattern, but GB2+ tumors tended to exhibit (while not significant) more frequently areas of glandular/papillary histology than WT ones (Table 2). Regarding metastasis, GB2+ mice and WT showed similar incidence of metastatic tumors (Table 2), whereas the number of metastatic foci was very variable within groups, and thus, no clear effect of the mouse genotype was found (Figure 6).

**TABLE 2:** Cancer incidence, histology, and frequency of metastasis in the R26-GB2/MMTV-NEU and R26-GB2/MMTV-PyMT models.

R26-GB2/MMTV-NEU model	WT	GB2+/-	GB2+/+	*p* value^a^	*p* value^b^
Breast cancer incidence	5/25 (20%)	12/26 (46%)	9/21 (43%)	0.11	0.04
Frequency of metastasis	3/5 (60%)	7/9 (78%)	7/9 (78%)	0.72	0.57
Tumor histology^d^				0.30	0.11
Solid pattern	3/5 (60%)	2/9 (20%)	1/9 (10%)		
Solid with necrosis	2/5 (40%)	5/9 (60%)	5/9 (50%)		
Solid with glandular/papillary area	0/5 (0%)	2/9 (20%)	3/9 (30%)		
**R26-GB2/MMTV-PyMT model**	**WT**	**GB2+/+**	***p* value** ^**c**^		
Breast cancer incidence	17/17 (100%)	10/10 (100%)	ND		
Frequency of metastasis	13/17 (76%)	5/10 (50%)	0.20		
Tumor histology			0.29		
Solid pattern	3/17 (18%)	4/10 (40%)			
Solid with necrosis	2/17 (12%)	2/10 (20%)			
Solid with glandular/papillary area	12/17 (70%)	4/10 (40%)			

GSDMB2 Heterozygous (GB2+/-), homozygous (GB2+/+) and control (WT) animals.

a p value of Chi^2^ test comparing the three genotypes separately.

b p value of Fisher’s exact test comparing either WT *vs*. GB2 (+/- and +/+ combined).

c p value of Fisher’s exact test comparing WT *vs.* GB2+/+.

d In three GB2+/-mice tumor histology could not be performed due to tissue degradation. ND, not done.

In the R26-GB2/MMTV-PyMT highly aggressive and fast-growing tumor model, no differences in tumor incidence, cancer latency, or histology were detected between GB2+/+ and WT mice (Table 2). WT and GB2+/+ animals had similar number of tumors (WT mean 5.9 tumors/mouse; GB2+/+ 5.3 tumors), total tumor weight (considering all cancers in each mouse), and cancer proliferation rate (Figure 6). Neither lung cancer incidence (Table 2) nor the metastatic foci number were significantly different between groups (Figure 6).

Therefore, consistent with the pro-cancer effect of GSDMB in human HER2-positive carcinomas (Hergueta-Redondo et al., 2016; Molina-Crespo et al., 2019), the results in murine models show that GSDMB2 promotes tumor incidence specifically in the context of HER2/NEU-driven carcinogenesis. Nonetheless, no strong effect of GSDMB2 on tumor growth, histology and metastatic potential was evidenced in any of the murine models analyzed.

### Analysis of other Histopathological Alterations (non-cancer) in the R26-GB2 model

Finally, apart from cancer, GSDMB function has been implicated in the response to infection with *Shigella* enterobacteria (Hansen et al., 2021) and in the pathobiology of diverse inflammatory diseases, including asthma, inflammatory bowel disease or arthritis, among others (Li et al., 2012; Morrison et al., 2013; Söderman et al., 2015; Panganiban et al., 2018; Chen et al., 2019; Rana et al., 2022). Therefore, we investigated if R26-GB2 mice exhibited any pathological (non-cancer) phenotype at the microscopic level. Lung pathologies (atelectasis or emphysema) tended to be more frequent in GB2 mice than WT (*p* = 0.06) (Supplementary Table S5). Moreover, our comprehensive analysis of multiple tissues detected infrequent pathological features in other organs but none of them associated significantly with GSDMB2 expression (Supplementary Table S5).

## Discussion

The GSDMs are functionally linked to multiple human diseases, including infectious, cardiovascular, neurodegenerative pathologies, chronic inflammatory conditions, deafness syndromes, and cancer, among others (Broz et al., 2020; Li et al., 2021; Liu et al., 2021). In fact, the GSDMs are promising therapeutic targets, with several GSDM-targeted compounds currently under pre-clinical evaluation (Liu et al., 2021; Ryder et al., 2021). Nonetheless, to decipher in detail the involvement of GSDMs in human pathologies *in vivo* there is an urgent need for the development of preclinical animal models, since to date very few Genetically Engineered Mouse Models (GEMM) have been generated. Interestingly, many *GSDM* KO models (*Gsdma1*, *Gsdmd*, *Gsdme*) show no spontaneous pathological phenotype (Fujii et al., 2008; Wang et al., 2017; Croes et al., 2019), but *GSDM in vivo* functions are unveiled only under particular stimuli and biological contexts. For example, *Gsdmd* KO mice do not show abnormalities (Fujii et al., 2008) but they are highly resistant to septic shock (pyroptosis-mediated) induced by Lipopolysaccharide injection (Kayagaki et al., 2015) and *Gsdme KO* mice develop normally but they are unresponsive to chemotherapy-induced systemic toxicity (Wang et al., 2017).

In cancer, human *GSDMB* has a complex role, and is considered either as a potential oncogene (frequently upregulated in cancer and promotes multiple pro-tumor functions) or tumor suppressor gene (GSDMB protein provokes cytotoxic anti-tumor effects) (Sarrió et al., 2021). However, two difficulties have prevented so far determining the precise GSDMB *in vivo* cancer functions: a) the lack of preclinical murine models, since mice and rat lack *GSDMB* orthologue; b) The existence of four GSDMB translated isoforms that can play different roles in cancer (Carl-McGrath et al., 2008; Sun et al., 2008; Hergueta-Redondo et al., 2014; Lutkowska et al., 2017) and inflammatory diseases (Morrison et al., 2013; Das et al., 2016; Panganiban et al., 2018).

Recently, two KI models of GSDMB isoform 1 (Hansen et al., 2021) or isoform 3 (Das et al., 2016) have been reported but, unfortunately, their effect in cancer development was not studied (discussed later). Here, we created the first GSDMB2 KI model, a transcript that increases invasiveness and metastatic potential in human breast cancers (Hergueta-Redondo et al., 2014), and after “whole-body” comprehensive analyses, we proved that GSDMB2 ubiquitous expression neither increases overall tumor development nor significantly affects the aggressiveness of spontaneous generated lung carcinomas. This demonstrates, for the first time, that GSDMB2 alone has no intrinsic tumor initiation capacity *in vivo*.

Nonetheless, in human breast and gastric cancers, GSDMB over-expression is frequently produced by the co-amplification of GSDMB and HER2/NEU within the 17q12-21 region (Saeki et al., 2009; Hergueta-Redondo et al., 2016), and indeed GSDMB upregulation associates with aggressive behavior, resistance to therapy and poor clinical outcome in HER2-positive breast carcinomas (Hergueta-Redondo et al., 2016; Molina-Crespo et al., 2019). Thus, we next tested if GSDMB2 pro-tumor *in vivo* functions required the pre-activation of HER2/NEU or other potent oncogenes, like the PyMT. Consistent with the GSDMB pro-tumor functions in human HER2 carcinomas, our two novel breast cancer models (R26-GB2/MMTV-NEU and R26-GB2/MMTV-PyMT) proved that GSDMB2 expression significantly augments breast cancer formation only in the context of HER2-driven tumorigenesis. Analyses by western blot and immunohistochemistry (using an antibody against the C-term HA tag) proved that full-length GSDMB2 was equally expressed in both models, demonstrating that the distinct effect in tumor development was not due to different levels of GSDMB overexpression but the biological context. Moreover, confirming our *in vitro* and/or *in vivo* data in human breast cancer cell lines and biopsies (Hergueta-Redondo et al., 2016; Molina-Crespo et al., 2019) GSDMB levels did not produce a clear effect on tumor weight or proliferation in our murine models. However, unlike human HER2 breast carcinomas and xenografted MCF7 cells, GSDMB upregulation did not significantly associate with increased metastatic potential. It should be noted that in the murine breast cancer models used, cancer cells only metastasize to the lungs, while in humans, metastatic locations are more diverse.

Overall, our breast cancer models can help to get novel mechanistic insights of the context-dependent role of GSDMB in cancer, and the R26-GB2/MMTV-NEU mice can be potentially used in the future to test novel oncologic treatments in HER2-positive tumors. Moreover, our results confirm previous studies in other GSDMs highlighting the relevance of the experimental setting to unveil the diverse, and sometimes opposing, roles of GSDM in tumors. For instance, human *GSDME*, broadly considered as tumor suppressor gene, reduces xenograft tumor growth in immunocompetent but not in immunodeficient mice (Rogers et al., 2019; Zhang et al., 2020). Additionally, *Gsdme* KO mice exhibit no clear effects on carcinogenesis, tumor differentiation and progression in two experimental models of intestinal cancer (the chemical induction by azoxymethane -AOM- or crossing with the Apc1638N/+ intestinal cancer mouse strain) (Croes et al., 2019), but these animals have significantly reduced colitis severity and tumor formation when AOM was combined with Dextran Sodium Sulphate (Tan et al., 2020).

Likewise, contrary to its pro-tumor effects, GSDMB can have tumor suppressor function in certain conditions (Zhou et al., 2020). Specifically, while exogenous *GSDMB* over-expression into two aggressive murine cancer xenografts models did not affect tumor progression, the treatment with PD-1 immune checkpoint inhibitors activated the GSDMB intrinsic pyroptotic activity in tumor cells through a non-cell autonomous mechanism mediated by NK and CD4^+^ T cells (Zhou et al., 2020). Only upon immune activation, mouse immunocytes released GZMA that cleaved human GSDMB, thus indicating that triggering an endogenous tumor reduction *in vivo* via GSDMB-mediated pyroptosis is not spontaneous and requires additional signals. Remarkably, consistent with GSDMB tumor suppressor function, Zhou and colleagues report a frequent GSDMB downregulation in gastroesophageal carcinomas, which contradicts previous studies in these tumors (Saeki et al., 2009, 2015; Komiyama et al., 2010).

In this sense, we observed that four (17%) WT animals and no GB2 mice of the R26-GB2 model developed macroscopic gastric carcinomas, while no differences in premalignant gastric lesions were observed between the genotypes. Unfortunately, the mechanism by which GSDMB2 might reduce gastric carcinomas could not be explored in our mice as we did not obtain any GSDMB2-positive gastric cancer. A potential GSDMB-mediated immune rejection of gastric tumor cells, as described above, is unlikely in our model since GSDMB2 is expressed in diverse cell types, including the immunocytes. In fact, it is possible that the immune recognition and stimulation of the anti-tumor response may be difficult to achieve in transgenic animals where the tumor and the surrounding cells carry the same genetic modifications, like our models and the *Gsdme* KO mice (Croes et al., 2019).

Moreover, the implication of GSDMB in gastric carcinogenesis is intricate, since normal and neoplastic cells differ in the usage of the two *GSDMB* gene promoters (LTR-derived and cellular promoter). This can lead to differential expression of GSDMB isoforms and total protein levels between healthy and cancerous stomach tissue (Sin et al., 2006; Komiyama et al., 2010). Therefore, to evaluate further the functional role of GSDMB in gastric cancer requires future studies expressing each of the isoforms under the recently described stomach-specific gene promoters (Seidlitz et al., 2019). Besides, it would be also necessary crossing the distinct *GSDMB* mice with GEMMs that develop gastric carcinomas (Jiang and Yu, 2017).

In addition to the effect of GSDMB on cancer, our study offers other interesting results. We demonstrated that GSDMB2 exhibits differential nuclear and/or cytoplasmic staining patterns among healthy tissues/organs, that mostly resemble those in the corresponding human tissues (Carl-McGrath et al., 2008; Sun et al., 2008; Das et al., 2016; Hergueta-Redondo et al., 2016; Zhou et al., 2020). Moreover, in spontaneous lung carcinomas (R26-GB2) and oncogene-driven breast cancers, GSDMB2 was mostly cytoplasmic but nuclear localization was noted in some tumor areas (usually more intense in GB2+/+ than GB2+/-mice, indicating enhanced protein accumulation with increased *GSDMB2* gene dosage). All these data suggest that this protein may have distinct biological functions depending on the cellular context and intracellular localization. GSDMB possesses a putative nuclear localization signal (residues 242–261) that is present in all GSDMB isoforms, and mutation/deletion of this sequence excludes GSDMB from the nucleus (Sun et al., 2008; Das et al., 2016). To date, the mechanism of GSDMB nucleus-cytoplasm shuttling is unknown, but nuclear GSDMB can regulate gene transcription. Indeed, nuclear GSDMB provokes the transcriptional induction of the same set of genes (TGF-β1 and 5-lipoxygenase) in human bronchial epithelial cells and the lungs of the hGSDMB^Zp3−Cre^ KI model (ubiquitously expressing the longest isoform, GSDMB3) after asthma challenge (Das et al., 2016). Transcriptional regulation by GSDMB3 provoked an asthmatic phenotype associated with increased airway hyper-responsiveness and remodeling in GEMMs (Das et al., 2016). In this sense, our R26-GB2 model can be useful in future studies to assess whether GSDMB regulates specific genes in particular tissues/cell types from both healthy and cancer conditions.

In addition to gene regulation, and despite physiological differences between humans and mice, accumulating evidences prove that exogenous human GSDMB expressed (mostly in the cytosol) in mouse cells can interact with mouse proteins and maintain other important biological activities. For instance, the mouse proteases caspase-4 and granzyme A, two key regulators/effectors of GSDMB, produce similar biological effect on GSDMB than the corresponding human proteases (Chen et al., 2019; Zhou et al., 2020).

Therefore, based on the above evidences proving the preclinical validity of *GSDMB* mouse models, and the overall similarity in GSDMB expression patterns between human and mouse tissues observed in our R26-GB2 mice, we propose that this strain could be also useful for deciphering the implication of GSDMB in other diseases, such as response to pathogens (Hansen et al., 2021), and inflammatory pathologies (asthma, type-I diabetes, inflammatory bowel diseases, biliary cirrhosis, and rheumatoid arthritis) (Li et al., 2012; Morrison et al., 2013; Söderman et al., 2015; Panganiban et al., 2018; Chen et al., 2019; Rana et al., 2022). In fact, using GEMM models, only the mechanistic implication of GSDMB3 has been demonstrated in asthma, while the GSDMB bactericidal function was not validated in GSDMB1 KI mice (Hansen et al., 2021). Strikingly, the asthmatic phenotype of the hGSDMB^Zp3−Cre^ model was mechanistically mediated by the transcriptional regulation of certain genes (Das et al., 2016), not via a cell death mechanism, while in human lung cells asthma has been linked to the pyroptotic effect of specific GSDMB isoforms (Morrison et al., 2013; Das et al., 2016; Panganiban et al., 2018). This suggests different pathogenic effects of GSDMB variants in asthma. While we did not evaluate the presence of asthmatic phenotype in our R26-GB2 model, we observed that other lung pathologies (atelectasis and emphysema) were more frequent (*p* = 0.06) in GB2 mice.

Summarizing, future studies comparing the different GSDMB isoform GEMMs would be required to identify the precise functions of each GSDMB variant in cancer and other diseases.

## Conclusions

The phenotypic characterization of our three novel GEMMs proves that GSDMB2 pro-tumor function is dependent on the biological context: GSDMB2 alone does not have strong *in vivo* spontaneous oncogenic properties (though a potential reduction of gastric tumorigenesis requires future studies), while in the mammary gland, it fuels carcinogenesis in concert with the HER2, and not PyMT, oncogene. Our GEMMs not only validate *in vivo* the role of GSDMB in human HER2-positive breast cancer but can also be useful for the future development of other tissue-specific and context-dependent disease models.

## Data Availability

The datasets presented in this study can be found in online repositories. The names of the repository/repositories and accession number(s) can be found in the article/Supplementary Material.
