# Supplementary material for: Gasdermin-B Pro-Tumor Function in Novel Knock-in Mouse Models Depends on the in vivo Biological Context
Source: Front Cell Dev Biol. 2022 Feb 24;10:813929. doi: 10.3389/fcell.2022.813929 (PMC8907722; doi:10.3389/fcell.2022.813929)
Supplement: Supplementary file 1 [file DataSheet1.PDF]

SUPPLEMENTARY DATA (Sarrio D et al): TABLES S1-S5 AND FIGURES S1-S4

Table S1: Primers and PCR conditions for genotyping

PRIMERS	bp	PCR conditions
**Transgene insertion into ROSA26 (3'arm)**<br>3'DV1-F1: 5'-GAACGGCATCAAGGTGAAC-3'<br>3'DV1-R1: 5'-ATCTCGAAGACCTGTTGCTG-3'	4800	94ºC 1min; (95ºC 30s, 64ºC 30s, 68ºC 5min) X40; 72ºC 7min.<br>LATaq Polymerase (Takara)
**Transgene insertion into ROSA26 (5'arm)**<br>NeoF3: 5'-CCAGTCATAGCCGAATAGCC-3'<br>5’DV1-F2: 5'-TAGGTAGGGGATCGGGACTC-3'	1800	94ºC 1min; (94ºC 30s, 68ºC 150s) X5; (95ºC 30s, 64ºC 30s, 68ºC 135s) X 35; 72ºC 7min.<br>LATaq Polymerase (Takara)
**CRE**<br>CreF: 5'-GCCTGCATTACCGGTCGATGC -3'<br>CreR: 5'-CAGGGTGTTATAAGCAATCCCC -3'	430	94ºC 5min; (94ºC 30s, 60ºC 30s, 72ºC 30s) X35; 72ºC 5min.<br>NZYTaq II Polymerase (NZYTech)
**Excision of NEO cassette & GSDMB2 activation**<br>GS-CreF: 5'-TATCAGTAAGGGAGCTGCAGTG-3'<br>GS-CreR3: 5'-TGAGGCCTGTTGTGTAGTGC-3'	700	94ºC 5min; (94ºC 30s, 60ºC 1 min, 72ºC 1min) X35; 72ºC 2min.<br>NZYTaq II Polymerase (NZYTech)
**ROSA26 WT allele**<br>ROSA26-F: 5'-TATCAGTAAGGGAGCTGCA -3'<br>ROSA26-R: 5'-ACCCCAGATGACTACCTATCC -3'	300	94ºC 5min; (94ºC 30s, 62ºC 30s, 72ºC 30s) X35; 72ºC 5min.<br>NZYTaq II Polymerase (NZYTech)
**GSDMB2-HA cDNA**<br>G2HA-F: 5'-TGGGTTCGGAGGATTCCAGA-3<br>G2HA-R: 5'-AGCATAATCAGGAACATCATACGG-3'	548	94ºC 5min; (94ºC 30s, 64ºC 30s, 72ºC 40s) X35; 72ºC 5min.<br>NZYTaq II Polymerase (NZYTech)
**GFP cDNA**<br>GFP-F: 5'-AAGGACGACGGCAACTACAAG-3'<br>GFP-R: 5'-AGGTAGTGGTTGTCGGGCAG-3'	300	94ºC 5min; (94ºC 30s, 68ºC 30s, 72ºC 30s) X35; 72ºC 5min.<br>NZYTaq II Polymerase (NZYTech)
**NEU (Rat)**<br>NEU F: 5'-CCCGAGTGTCAGCCTCAAA-3'<br>NEU R: 5'-GCAGGCTGCACACTGATCA-3'	600	94ºC 5min; (94ºC 30s, 64ºC-0,5ºC 35s, 72ºC 40s) X12 (94ºC 30s, 58ºC 35s, 72ºC 40s) X25 72ºC 5min. Taqpol (Biotools)
**PYMT**<br>POL F: 5'-ATCGGGCTCAGCAACACAAG-3'<br>POL R: 5'-AACGGCGGAGCGAGGAACTG-3'	280	94ºC 5min; (94ºC 30s, 61ºC 30s 72ºC 30s) X30 72ºC 5min.<br>NZYTaq II Polymerase (NZYTech)

Tissue	GSDMB2-HA staining (intensity and localization) in R26-GB2 mice	GSDMB staining in human tissues (reference)
**Skin (tail)**	Strong nuclear and cytoplasmic in epidermis, hair follicles and sebaceous glands.	Not previously analyzed
**Esophagus**	Strong nuclear staining in the squamous epithelium.	Cytoplasmic and nuclear expression in the squamous epithelium (Protein Atlas and Zhou et al, 2021)
**Stomach**	Moderate cytoplasmic staining in the glandular epithelium.	Medium expression and cytoplasmic in the glandular epithelium (Zhou et al, 2021). Moderate expression and mostly cytoplasmic in epithelia (Protein Atlas). Cytoplasmic and vesicular staining mostly apical surface of gastric epithelium (McGrath et al, 2008).
**Intestine**	Strong cytoplasmic staining and focal nuclear localization in the epithelium.	Moderate expression cytoplasmic and focally nuclear (Zhou et al 2021). Medium-high cytoplasmic staining, focally nuclear in enterocytes (Protein Atlas). Cytoplasmic and vesicular staining mostly apical and luminal surface of the epithelium (McGrath et al, 2008).
**Liver**	Weak cytoplasmic staining in hepatocytes.	Weak diffuse cytoplasmic staining in hepatocytes (Sun et al 2008 and Protein Atlas). Cytoplasmic and vesicular staining mostly apical surface in hepatocytes (McGrath et al, 2008)
**Pancreas**	Moderate cytoplasmic staining in pancreatic cells.	Moderate cytoplasmic staining in endocrine and exocrine pancreas (Protein Atlas).
**Kidney**	Weak cytoplasmic in glomeruli and tubules. Strong cytoplasmic in renal papilla.	Weak diffuse cytoplasmic staining in tubules (Protein Atlas).
**Lung**	Weak cytoplasmic and focal nuclear staining in bronchus/bronchioles.	Cytoplasmic and focal nuclear staining in bronchial cells (Das et al, 2016). Cytoplasmic and focal nuclear in bronchus (Protein Atlas)
**Heart**	Weak cytoplasmic staining in muscle cells.	Weak cytoplasmic staining in cardiomyocytes (Protein Atlas)
**Brain**	Weak cytoplasmic and focal nuclear overall. Strong cytoplasmic in ependymal cells of choroid plexus.	Medium expression, cytoplasmic staining and focally nuclear in granular and Purkinje cells. Nuclear in cortical neurons (Protein Atlas)
**Breast**	Moderate cytoplasmic staining in mammary gland epithelia.	Weak cytoplasmic in epithelial cells (Protein atlas)
**Salivary gland**	Weak cytoplasmic in glandular cells.	Not analyzed
**Spleen**	Weak diffuse cytoplasmic staining in lymphoid cells.	Cytoplasmic staining in red pulp lymphoid cells (Protein atlas).
**Uterus**	Strong cytoplasmic and focal nuclear expression in cervix epithelium.	Strong cytoplasmic and nuclear expression in cervix epithelium (Sun et al, 2008). Cytoplasmic and nuclear expression in the epithelium (Zhou et al 2021). Weak cytoplasmic staining in endometrium (Protein Atlas)
**Testicles**	Very strong cytoplasmic and nuclear staining in seminiferous tubules. Weak cytoplasmic in epididymis.	Medium-high expression cytoplasmic and focally nuclear staining in seminiferous ducts and Leydig cells. No expression in epididymis (Protein Atlas)

Table S2: Immunohistochemical expression and intracellular localization of GSDMB2-HA in selected tissues from the R26-GB2 mouse model and in corresponding human tissues (data from other sources). In the Protein Atlas database the most frequent and strongest staining pattern within all analyzed antibodies was selected.

Table S3. Histological characteristics of the spontaneous tumors originated in GSDMB2-HA knock-in model (R26-GB2) and control (WT) mice.

TUMOR HISTOLOGY	WT	GB2+/-	GB2+/+	P value[1]	P value[2]
**Lung Adenocarcinoma (n)**	**9**	**17**	**5**	0.69	0.59
Well differentiated	5 (56%)	11 (65%)	2 (40%)		
Moderately differentiated	2 (22%)	5 (29%)	2 (40%)		
Poorly differentiated	2 (22%)	1 (6%)	1 (20%)		
**Gastric carcinoma (n)**	**4**	**0**	**0**	ND	ND
Low grade	3 (75%)	0	0		
High grade	1 (25%)	0	0		

GSDMB2 Heterozygous (GB2+/-), homozygous (GB2+/+) and control (WT) animals were generated by crossing parental heterozygous mice. [1] p value of Chi^2 test comparing the three genotypes separately; [2] p value of Fisher's exact test comparing WT vs GB2 (+/- and +/+ combined). ND, not done

Table S4. Pre-malignant microscopic lesions in lungs and stomach from GSDMB2-HA knock-in model (R26-GB2) and control (WT) mice.

Lesion	WT	GB2+/-	GB2+/+	P Value[1]	P Value[2]
Gastric adenomas and polyps	1/7 (14%)	0/13 (0%)	1/10 (10%)	0.4	0.41
Chronic gastritis	3/7 (43%)	4/13 (30%)	3/10 (30%)	0.8	0.36
Lung adenomatous hyperplasia	1/11 (9%)	0/13 (0%)	2/14 (14%)	0.4	0.99

GSDMB2 Heterozygous (GB2+/-), homozygous (GB2+/+) and control (WT) animals were generated by crossing parental heterozygous mice. [1] p value of Chi^2 test comparing the three genotypes separately; [2] p value of Fisher's exact test comparing WT vs GB2 (+/- and +/+ combined).

Table S5. Frequency of other non-cancer microscopic lesions from GSDMB2-HA knock-in model (R26-GB2) and control (WT) mice.

Non-cancer lesion	WT	GB2+/-	GB2+/+	P Value[1]	P Value[2]
Lung Emphysema	1/11 (9%)	4/13 (31%)	1/14 (7%)	0.18	0.65
Lung Atelectasis	0/11 (0%)	2/13 (15%)	4/14 (29%)	0.15	0.15
Liver Steatosis or necrosis	3/5 (60%)	7/8 (87.5%)	3/6 (50%)	0.29	0.99
Uterine/ovarian benign cysts	0/6 (0%)	4/9 (45%)	1/5 (20%)	0.14	0.26
Other analyzed tissues with <2 cases of pathology *	27	28	27		

GSDMB2 Heterozygous (GB2+/-), homozygous (GB2+/+) and control (WT) animals were generated by crossing parental heterozygous mice.[1] p value of Chi^2 test comparing the three genotypes separately; [2] p value of Fisher's exact test comparing WT vs GB2 (+/- and +/+ combined). *: brain, salivary gland, heart, bladder, kidney, intestine, pancreas, spleen and male reproductive organs.

SUPPLEMENTARY FIG 1: UNCROPPED GELS FOR FIGURE 1

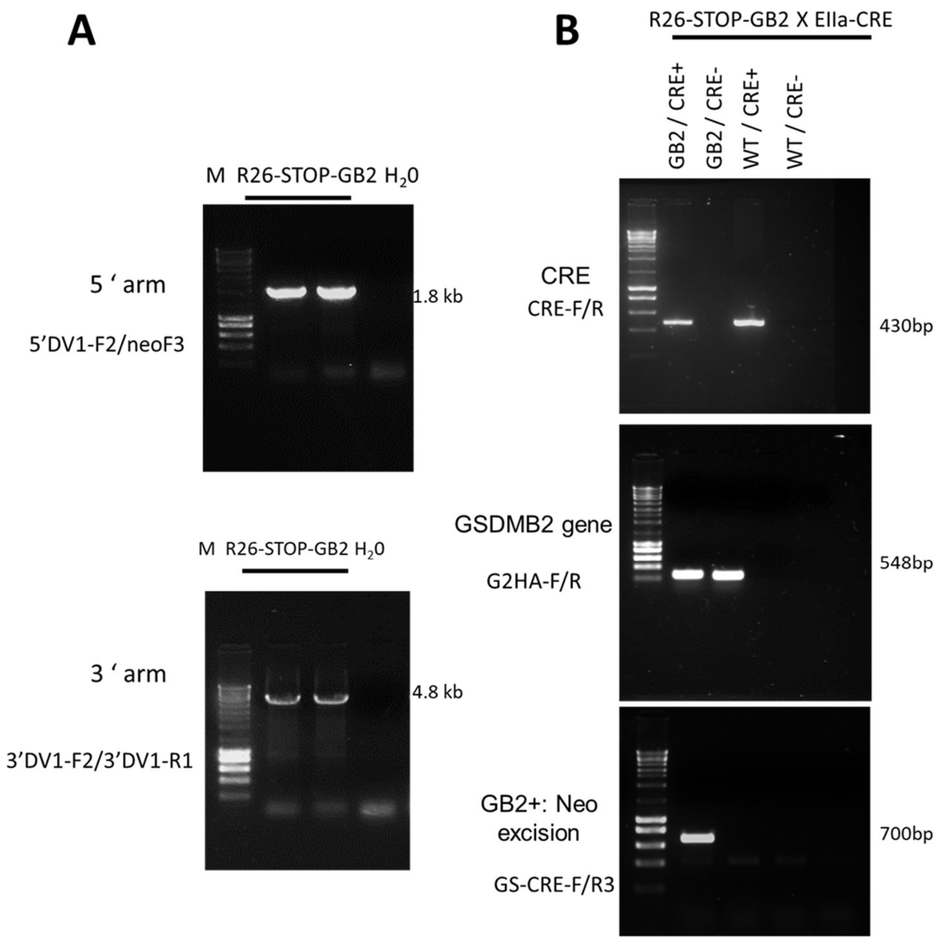

SUPPLEMENTARY FIG 1: UNCROPPED BLOTS FOR FIGURE 2 (PANEL A, TOP)

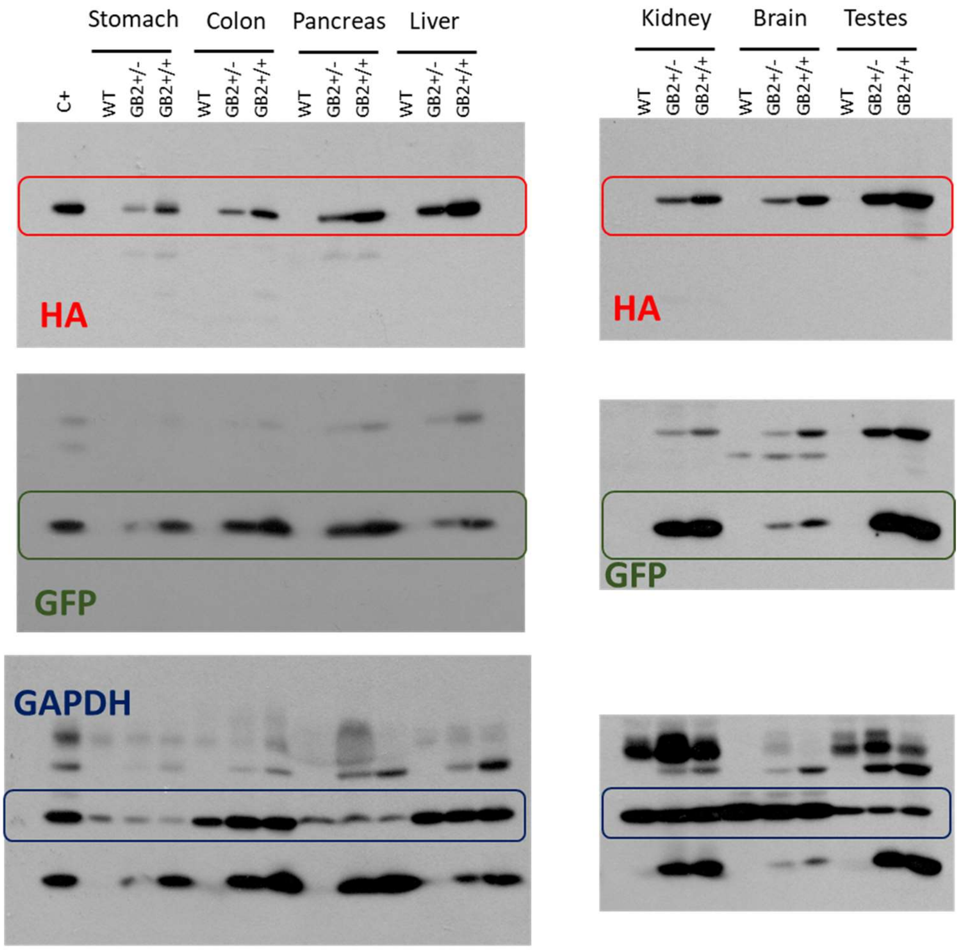

Cropped areas are indicated with color boxes

SUPPLEMENTARY FIG 1: UNCROPPED BLOTS FOR FIGURE 2 (PANEL A, BOTTOM)

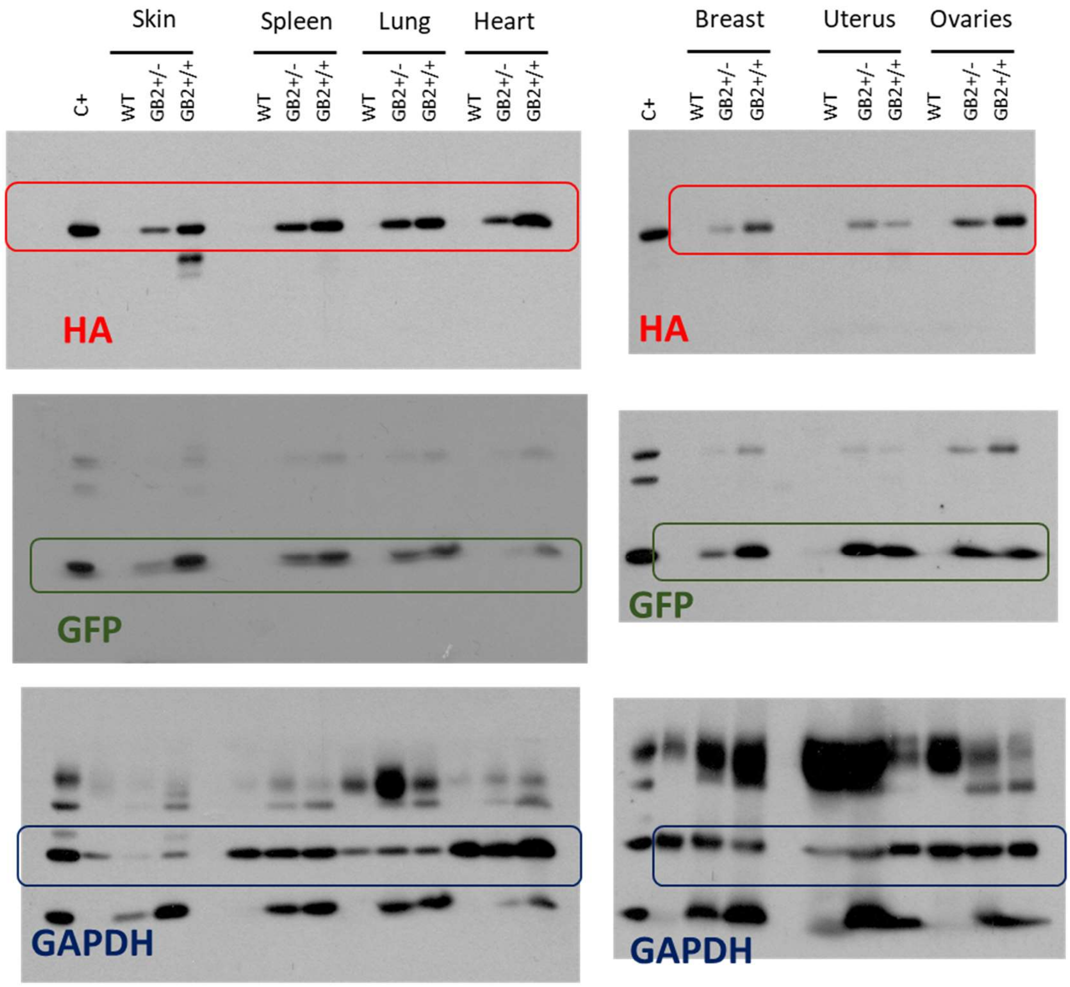

UNCROPPED BLOTS FOR FIGURE 2 (PANEL B)

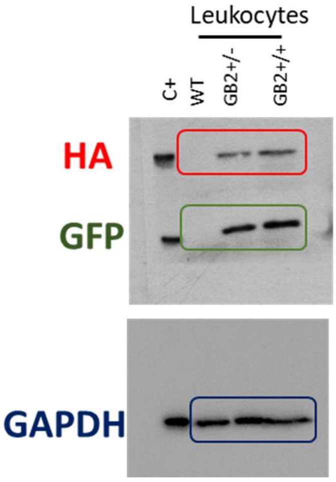

Cropped areas are indicated with color boxes

SUPPLEMENTARY FIG 1: UNCROPPED BLOTS FOR FIGURE 5C

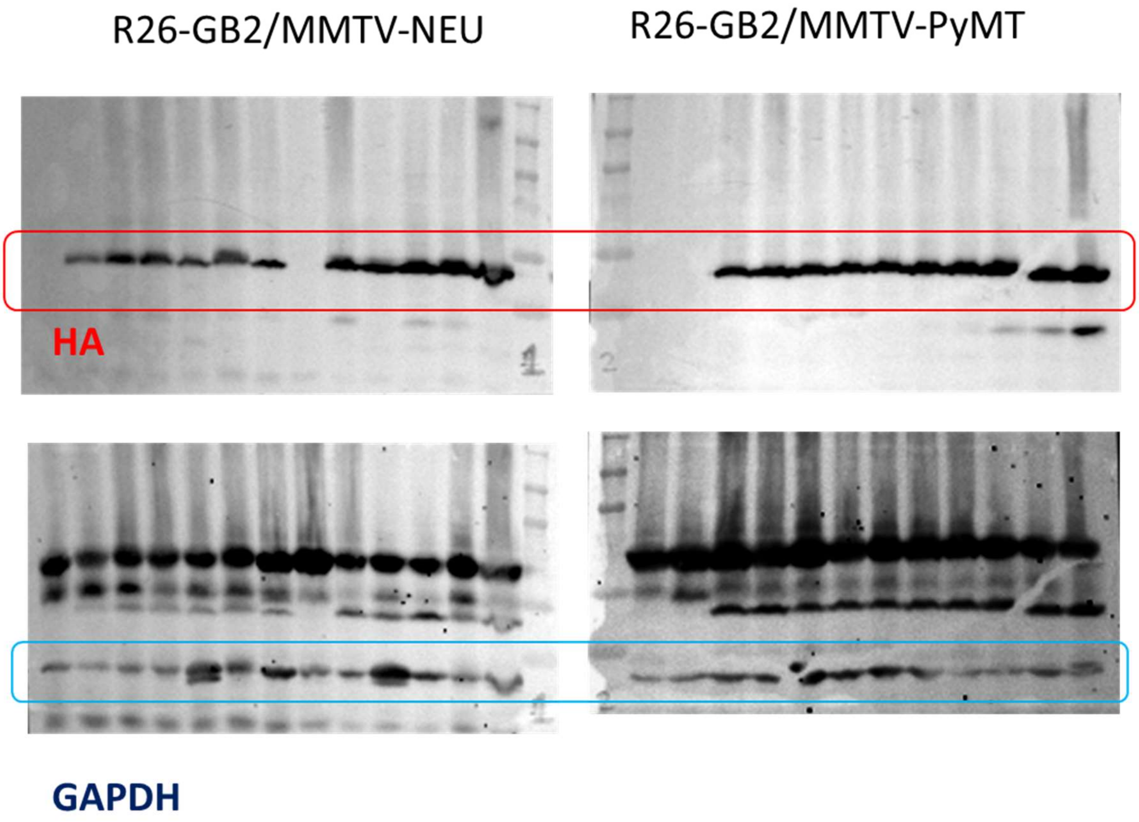

Cropped areas are indicated with color boxes

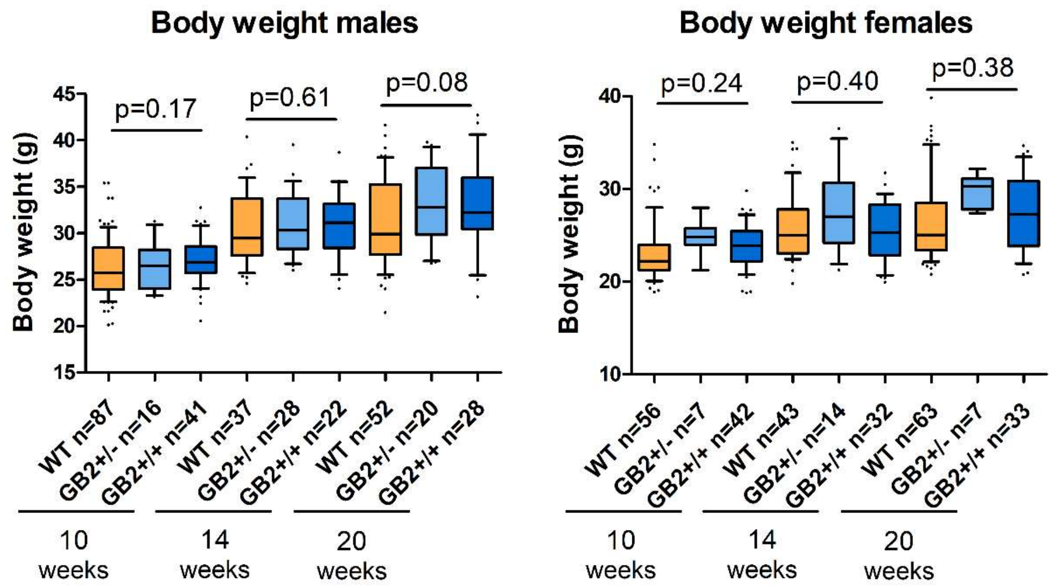

Supplementary Figure S2. Total body weight of R26-GB2 mice compared to WT controls. The age (in weeks) and the number of mice per group are indicated. Box plots represent median values and quartiles, and whiskers the 10-90 percentile. Heterozygous (GB2+/-), homozygous (GB2+/+) and control (WT) were generated by crossing parental heterozygous mice. P value of t-test comparing WT and GB2+/+.

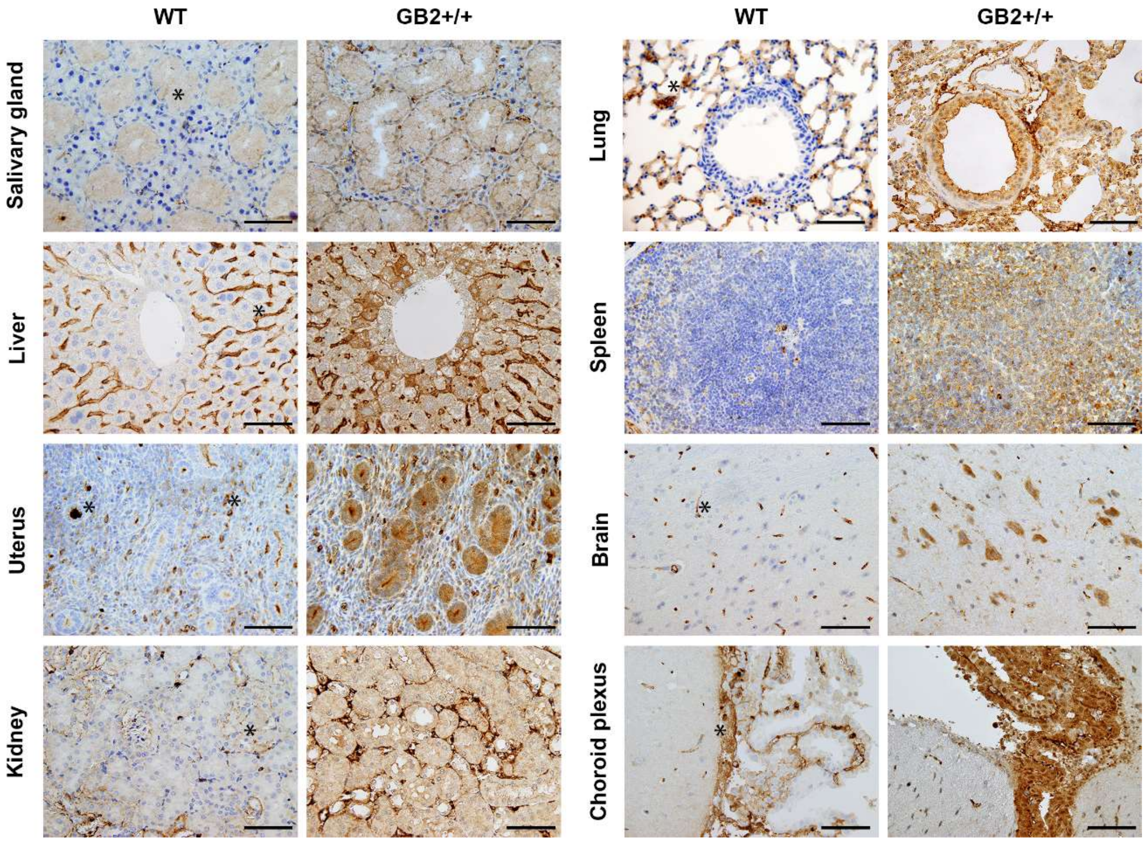

Supplementary Figure S3. Immunohistochemical expression of GSDMB2-HA in different tissues from the R26-GB2 mouse model. Representative images of tissues from homozygous (GB2+/+) and control (WT) mouse littermates. * Unspecific staining. Scale bar, 100 µm.

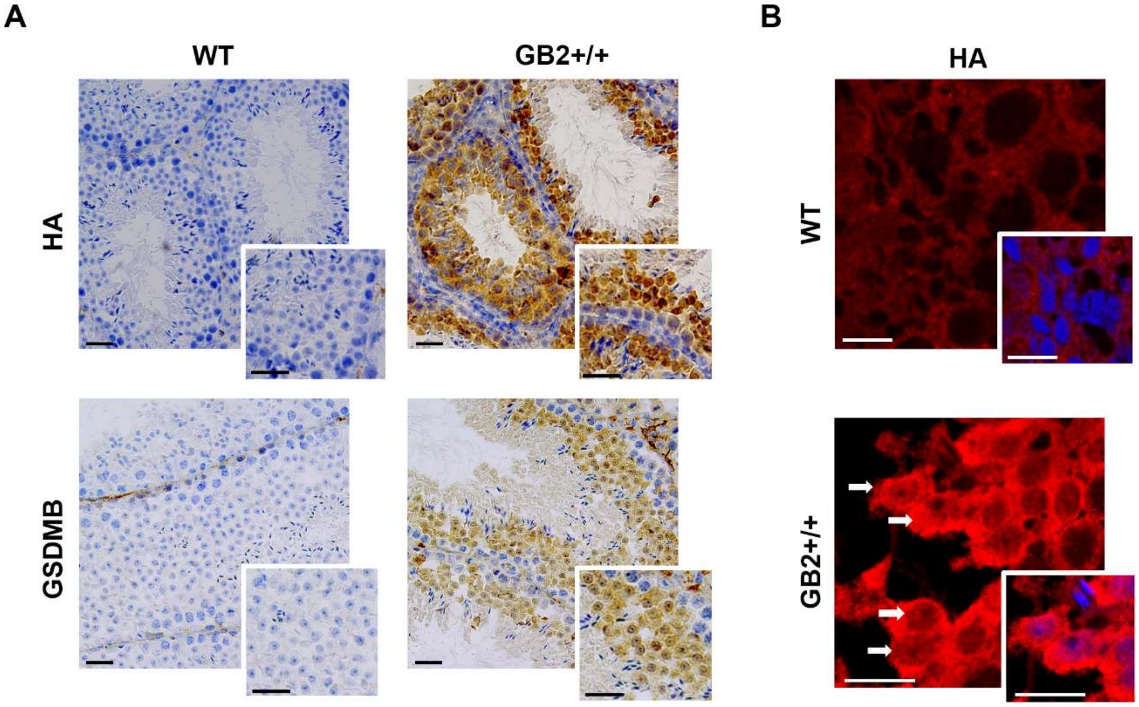

Supplementary Figure S4. Nuclear and cytoplasmic localization of GSDMB2-HA in the testes of R26-GB2 mice. **A:** Immunohistochemical expression using rat anti-HA antibody (top) and mouse anti-GSDMB antibody (Hergueta-Redondo et al 2016) in testes from WT and homozygous (GB2 +/+) mice. Inset: zoomed images. Scale bar 50 µm. **B:** Immunofluorescence staining and confocal imaging of GSDMB2-HA in the samples depicted in (A). GSDMB2-HA (red) localizes focally in the cell nucleus (arrows). In the inset images, nuclei are stained with DAPI (blue). Scale bar 25 µm
